# Disruption of STAT3 signalling promotes KRAS-induced lung tumorigenesis

**DOI:** 10.1038/ncomms7285

**Published:** 2015-03-03

**Authors:** Beatrice Grabner, Daniel Schramek, Kristina M. Mueller, Herwig P. Moll, Jasmin Svinka, Thomas Hoffmann, Eva Bauer, Leander Blaas, Natascha Hruschka, Katalin Zboray, Patricia Stiedl, Harini Nivarthi, Edith Bogner, Wolfgang Gruber, Thomas Mohr, Ralf Harun Zwick, Lukas Kenner, Valeria Poli, Fritz Aberger, Dagmar Stoiber, Gerda Egger, Harald Esterbauer, Johannes Zuber, Richard Moriggl, Robert Eferl, Balázs Győrffy, Josef M. Penninger, Helmut Popper, Emilio Casanova

**Affiliations:** 1 Ludwig Boltzmann Institute for Cancer Research (LBI-CR), Vienna 1090, Austria; 2 Institute of Molecular Biotechnology of the Austrian Academy of Sciences (IMBA), Vienna 1030, Austria; 3 Institute of Animal Breeding and Genetics, University of Veterinary Medicine, Vienna 1210 and Medical University Vienna, Vienna 1090, Austria; 4 Institute of Pharmacology, Medical University of Vienna, Vienna 1090, Austria; 5 Institute of Cancer Research & Department of Internal Medicine I, Comprehensive Cancer Center, Medical University of Vienna, Vienna 1090, Austria; 6 Research Institute of Molecular Pathology (IMP), Dr. Bohr Gasse 7, Vienna 1030, Austria; 7 Department of Biosciences and Nutrition, Center for Innovative Medicine, Karolinska Institutet, Novum, Huddinge 141 83, Sweden; 8 Department of Molecular Biology, Paris-Lodron University of Salzburg, Salzburg 5020, Austria; 9 Department of Internal Medicine, Karl Landsteiner University, 3430 Tulln, Austria; 10 Clinical Institute of Pathology, Medical University of Vienna, Vienna 1090, Austria; 11 Unit of Pathology of Laboratory Animals (UPLA), University of Veterinary Medicine Vienna, 1210 Vienna, Austria; 12 Molecular Biotechnology Center (MBC), Department of Genetics, Biology and Biochemistry, University of Turin, Turin 10126, Italy; 13 Department of Laboratory Medicine, Medical University of Vienna, Vienna 1090, Austria; 14 MTA TTK Lendület Cancer Biomarker Research Group, Budapest 1117, Hungary; 15 2nd Department of Pediatrics, Semmelweis University, Budapest 1094, Hungary; 16 MTA-SE Pediatrics and Nephrology Research Group, Budapest 1083, Hungary; 17 Institute of Pathology, Research Unit Molecular Lung and Pleura Pathology, Medical University of Graz, Graz 8036, Austria; 18 Department of Physiology, Center of Physiology and Pharmacology, Comprehensive Cancer Center, Medical University of Vienna, Vienna 1090, Austria

## Abstract

STAT3 is considered to play an oncogenic role in several malignancies including lung cancer; consequently, targeting STAT3 is currently proposed as therapeutic intervention. Here we demonstrate that STAT3 plays an unexpected tumour-suppressive role in *KRAS* mutant lung adenocarcinoma (AC). Indeed, lung tissue-specific inactivation of *Stat3* in mice results in increased *Kras*^G12D^-driven AC initiation and malignant progression leading to markedly reduced survival. Knockdown of STAT3 in xenografted human AC cells increases tumour growth. Clinically, low *STAT3* expression levels correlate with poor survival and advanced malignancy in human lung AC patients with smoking history, which are prone to *KRAS* mutations. Consistently, *KRAS* mutant lung tumours exhibit reduced STAT3 levels. Mechanistically, we demonstrate that STAT3 controls NF-κB-induced *IL-8* expression by sequestering NF-κB within the cytoplasm, thereby inhibiting IL-8-mediated myeloid tumour infiltration and tumour vascularization and hence tumour progression. These results elucidate a novel STAT3–NF-κB–IL-8 axis in *KRAS* mutant AC with therapeutic and prognostic relevance.

## Abstract (web summary)

STAT3 is an intracellular transducer of cytokine signals that cooperates with Ras in tumour formation and is often activated in lung cancer. Here the authors show that STAT3 acts as a tumour suppressor in a mouse model of Kras-driven lung adenocarcinoma.

## Graphical abstract

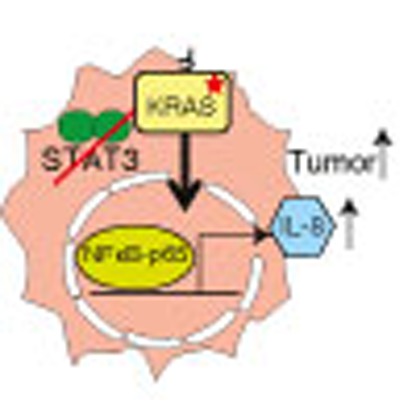

Lung cancer is still the leading cause of cancer deaths worldwide^1^. The most frequent genetic alterations found in lung adenocarcinomas (ACs) are missense mutations and amplifications of kirsten rat sarcoma viral oncogene (*KRAS)* and epidermal growth factor receptor *(EGFR)* in about 30% and 20% of all Caucasian cases, respectively^2^,^3^. Although several targeted anti-EGFR therapies are effective in patients with *EGFR* mutations, oncogenic *KRAS* is still considered as an undruggable target. To elucidate further treatment strategies, the focus has therefore shifted towards KRAS-cooperating and downstream signalling pathways. Signal transducer and activator of transcription 3 (*STAT3*) represents one important transcription factor thought to cooperate with *rat sarcoma viral oncogene (RAS)* mutations during tumorigenesis^4^,^5^. STAT3 is activated in response to several cytokines and growth factors, such as interleukin-6 (IL-6), oncostatin-M (OSM) and epidermal growth factor (EGF). Upon binding of the ligand to its cognate receptor, STAT3 becomes tyrosine-phosphorylated and forms homodimers or heterodimers before translocating into the nucleus to induce the transcription of several target genes implicated in cell cycle regulation, apoptosis, angiogenesis, tumour invasion and metastasis^6^. Depending on the oncogenic driver mutation or on the cell type, STAT3 has been reported to play either pro-oncogenic^4^,^5^,^7^ or tumour-suppressive roles *in vivo* and *in vitro*^8^,^9^,^10^,^11^,^12^. Interestingly, STAT3 was shown to be activated in 54% of lung AC patient samples and human lung cancer cell lines^13^,^14^,^15^. Furthermore, based on *in vitro* and xenograft models, STAT3 is thought to play a tumour-promoting role in non-small-cell lung cancer (NSCLC) and during acquired drug resistance^13^,^14^,^16^,^17^,^18^.

Here, we investigated the role of STAT3 during oncogenic *Kras*-driven lung tumorigenesis using the Cre-inducible Lox-Stop-Lox-*Kras*^*G12D/+*^ knock-in lung cancer mouse model^19^ and a human xenograft model. Surprisingly, our results show that STAT3 functions as tumour suppressor in *Kras*^G12D/+^ lung tumours as well as *KRAS* mutant human AC cell lines. Deletion of STAT3 in *Kras*^G12D/+^ lung tumours and human AC resulted in an increased tumour growth, higher tumour grade, increased vascularization, changes in the tumour microenvironment and significantly reduced survival. Mechanistically, we show that genetic ablation of *Stat3* in murine as well as knockdown of *STAT3* in human cells resulted in an increase of nuclear factor-kappa B (*NF-κB*)-induced expression of the pro-angiogenic chemokine ligand 1 (*CXCL1*; murine orthologue to the human *IL-8*), thereby promoting tumour growth. Pharmacological inhibition of CXCL1’s cognate receptor CXCR2 normalizes tumour vascularization and microenvironment and reduces tumour burden. Thus, STAT3 functions as tumour suppressor by sequestering NF-κB in the cytoplasm and thereby reducing NF-κB-dependent *CXCL1* transcription.

## Results

### STAT3 suppresses *Kras*-induced lung tumorigenesis

First, we investigated the activation status of STAT3 within murine *Kras*^*G12D*^-driven lung tumours. In all, 20–30% of tumour cells showed active STAT3 (tyrosine 705 phosphorylated STAT3) throughout tumour development. ELISA analysis revealed increased expression of STAT3-activating cytokines such as EGF, OSM and IL-6 in lungs harbouring *Kras*^*G12D*^-driven tumours compared with healthy control lungs (Supplementary Fig. 1a,b).

**Figure 1:**
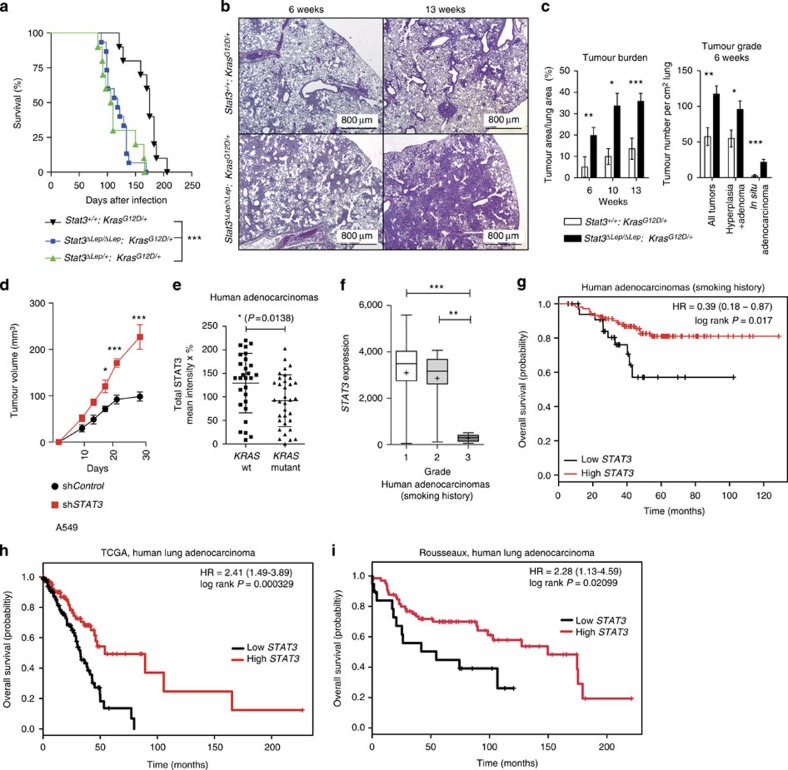
STAT3 suppresses *Kras*-induced lung tumorigenesis. (**a**) Kaplan–Meier plot showing overall survival of male mice with the indicated genotypes infected with AdCre (*n*≥10 male mice per genotype; log-rank test). (**b**) Representative haematoxylin and eosin stainings and quantification of tumour area/lung area in mice at indicated time points. Data were analysed by Student’s *t*-test and are shown as mean±s.d., *n*=6–10. Scale bar, 800 μm. (**c**) Tumour grade quantification at 6 weeks post AdCre infection in *Stat3*^*ΔLep/ΔLep*^*:Kras*^*G12D/+*^ compared with *Stat3*^*+/+*^*:Kras*^*G12D/+*^ mice. Data were analysed by Student’s *t*-test and are shown as mean±s.e.m., *n*≥7 mice per genotype. (**d**) Human A549 lung AC cells were transducted with scrambled shRNA (sh*Control*) or shRNA against *STAT3* (sh*STAT3*) and 2 × 10^6^ cells were injected into male nude mice (*n*=5 per group). Data were analysed by two-way analysis of variance with Bonferroni correction and are shown as mean±s.e.m. (**e**) IHC evaluation of total STAT3 in human mucinous adenocarcinomas with or without *KRAS* mutation. Intensity of staining (0/+1/+2/+3) was multiplied by percentage of stained tumour cells. Data are shown as mean±s.d. *n*≥28 per group. (Mann–Whitney *U*-test, *P*=0.0138) (**f**) *STAT3* mRNA expression of lung adenocarcinoma patients harbouring smoking history at different tumour grades is shown (*n*=139; grade I, low metastatic potential; grade II, intermediate metastatic potential; grade III, high metastatic potential^25^). (Kruskal–Wallis test with Dunn’s multiple comparison testing *P*<0.0001) (**g**) Kaplan–Meier plot showing overall survival of lung adenocarcinoma patients with smoking history stratified by high or low *STAT3* mRNA expression (lower quartile, log-rank) *n*≥35–104 per group. Kaplan–Meier plot showing overall survival of the (**h**) TCGA cohort or the (**i**) Rousseaux cohort stratified by high or low STAT3 mRNA expression (log-rank test). For all graphs: *P<0.05; **P<0.01; ***P<0.001.

To functionally test the role of STAT3 during *Kras*^*G12D*^-driven lung tumour formation, we crossed the *Kras*^*LSL-G12D/+*^ strain^19^ with conditional *Stat3*^fl/fl^ mice^20^. Lung epithelial-specific activation of oncogenic *Kras*^G12D^ and concomitant deletion of *Stat3* was achieved by adenoviral delivery of Cre-recombinase (AdCre) through inhalation of 8- to 10-week-old *Stat3*^fl/fl^:*Kras*^*LSL-G12D/+*^ mice (*Stat3*^*ΔLep/ΔLep*^:*Kras*^*G12D*/+^ hereafter; ^*Δ*Lep^: deleted in the lung epithelium). Efficient deletion of *Stat3* in lung tumour cells was confirmed by immunohistochemistry (IHC) of total STAT3 (Supplementary Fig. 1c). Notably, genetic ablation of *Stat3* resulted in a markedly shortened survival. Although *Stat3*^*ΔLep/ΔLep*^:*Kras*^*G12D/+*^ males showed a median survival of 118 days post AdCre infection, the control *Stat3*^+/+^:*Kras*^*G12D/+*^ male littermates survived 175 days (Fig. 1a). Interestingly, heterozygous loss of STAT3 within the tumours (*Stat3*^*ΔLep/+*^:*Kras*^*G12D/+*^) led to the same drastic phenotype as observed in *Stat3*^*ΔLep/ΔLep*^:*Kras*^*G12D/+*^ animals with a median survival of 108 days. Of note, female *Stat3*^*ΔLep/ΔLep*^:*Kras*^G12D/+^ mice showed a less pronounced survival disadvantage when compared with their female littermate controls (Supplementary Fig. 1d).

We performed tumour analysis in male mice at three different stages: 6, 10 and 13 weeks post AdCre-induction. *Stat3*^*ΔLep/ΔLep*^:*Kras*^*G12D/+*^ mice revealed a dramatically increased tumour burden compared with littermate controls at all time points (Fig. 1b). Already 6 weeks after tumour induction, *Stat3*^*ΔLep/ΔLep*^:*Kras*^*G12D*/+^ mice showed an increased number of hyperplasia (including multifocal pneumocyte hyperplasia and adenoma) as well as malignant *in situ* ACs compared with control *Kras*^*G12D*/+^ animals (Fig. 1c and Supplementary Fig. 1e). At 13 weeks post AdCre-induction, the size of ACs spreading throughout the whole lung precluded analysis of individual tumours. Therefore, we assessed tumour area/lung area and observed significantly increased areas of multifocal hyperplasia and *in situ* ACs in *Stat3*^*ΔLep/ΔLep*^:*Kras*^*G12D/+*^ mice compared with control animals (Supplementary Fig. 1e). Of note, small areas of invasive ACs were observed in both experimental groups at 13 weeks post AdCre inhalation (Supplementary Fig. 1e). In line with these observations, *Stat3*-deficient multifocal hyperplasia at 6 and 13 weeks as well as *in situ* ACs at 6 weeks post AdCre infection displayed significantly enhanced cell proliferation compared with tumours from *Stat3*-proficient control littermates (Supplementary Fig. 1f), whereas the low amount of apoptosis observed in the *Kras*^*G12D*^-driven lung tumours was not altered upon genetic ablation of STAT3 (refs 21, 22; Supplementary Fig. 1f). In addition, xenograft experiments using the human AC cell line A549, which carries a *KRAS* mutation in the same amino acid as our mouse lung cancer model (G12S), showed increased tumour growth upon *STAT3* knockdown, thus confirming the observations made in the mouse model (Fig. 1d and Supplementary Fig. 2a). Together, these data show that *STAT3* suppresses tumorigenesis of *Kras*^*G12D*^-driven lung tumours in mouse and human xenograft models.

Given these results, we hypothesized that human AC with a molecular signature similar to *Stat3*-deficient tumours might have a worse prognosis. To test this idea, microarray data were used to identify differentially expressed genes between *Stat3*-proficient and *Stat3*-deficient tumours isolated 13 weeks post induction. Subsequent gene set enrichment analysis (GSEA) revealed that *Stat3*^*ΔLep/ΔLep*^:*Kras*^*G12D/+*^ tumours indeed showed significant positive enrichment for genes associated with poor prognosis in human AC lung cancers and, conversely, significant negative enrichment for genes associated with good prognosis in humans (Supplementary Fig. 1g; ref. 23).

These data show that *Stat3*-deficient tumours share a transcriptional profile with advanced progression and poor prognosis observed in human AC. In addition, the gene expression profile of murine *Kras*^*G12D*^ tumours devoid of STAT3 signalling significantly overlapped with human *KRAS* mutant lung AC, and, conversely, murine *Kras*^*G12D*^ tumours competent of STAT3 signalling showed a significant enrichment for genes usually downregulated in human *KRAS* mutant lung AC (Supplementary Fig. 1h; ref. 24). This result led us to test whether *STAT3* signalling is perturbed in human lung ACs and we analysed four different patient cohorts. Interestingly, we found a significant decrease in *STAT3* expression levels in *KRAS* mutant human tumours compared with lung tumours with wild-type *KRAS* in the first cohort of smoking patients (Fig. 1e). STAT3 activation status was significantly reduced when compared with an *EGFR* mutant cohort (Supplementary Fig. 2b). Furthermore, analysis of the second cohort containing 139 patients with known smoking history showed that *STAT3* expression is significantly reduced in grade III tumours (with high metastatic potential^25^; Fig. 1f, *P*=<0.0001)^26^. Furthermore, in this patient cohort we found that low *STAT3* expression levels correlate with reduced overall survival (Fig. 1g, *P*=0.017)^26^. Of note, it is well established that smoking is strongly associated with *KRAS* mutations^3^. In this line, *STAT3* expression levels do not stratify AC patients without smoking history, a subgroup less prone to harbour *KRAS* mutations^3^ (Supplementary Fig. 2c). The third and fourth cohort containing 255 and 85 lung AC patient samples, respectively, confirmed our initial findings that *STAT3* expression levels are significantly reduced in the high-risk population (Supplementary Fig. 2d and e)^27^. Furthermore, low *STAT3* expression correlates with poor overall survival outcome in lung AC patients (Fig. 1h, *P*=0.000329; Fig. 1i, *P*=0.002099). Thus, these results indicate that high-grade tumours as well as lung AC are selected for low *STAT3* expression presumably to overcome *STAT3*-mediated tumour suppression, which is consistent with poor survival observed in patients with low levels of *STAT3* and a smoking history.

### STAT3 alters tumour microenvironment and angiogenesis

**Figure 2:**
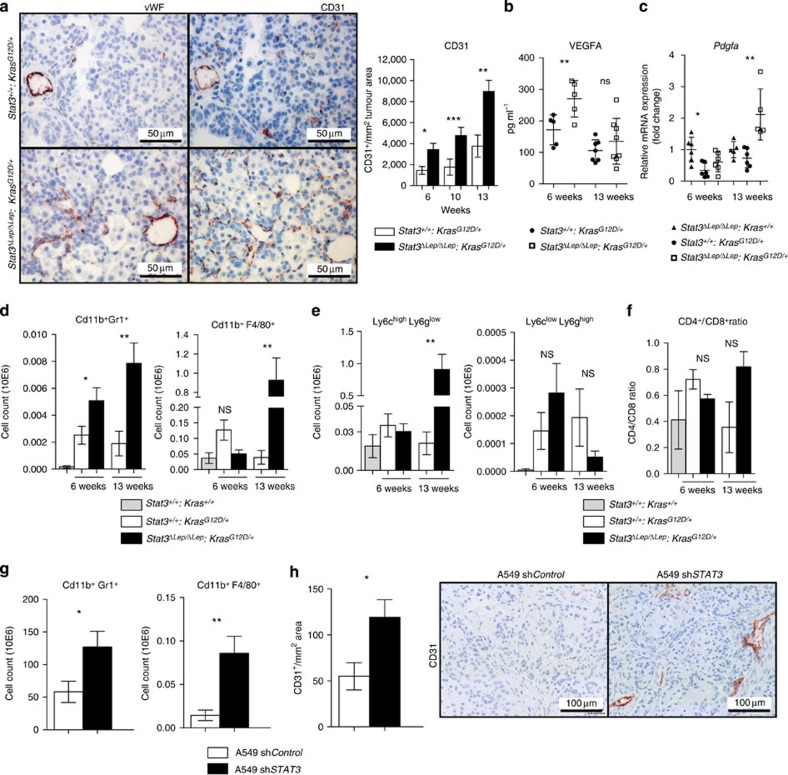
STAT3 alters tumour microenvironment and angiogenesis. (**a**) IHC analysis of von Willebrand Factor (vWF) and CD31 is shown in consecutive sections of murine lung tumours of the indicated genotype. On right, CD31^+^ counts per tumour area (mm^2^) quantification at indicated time points. Data were analysed by Student’s *t*-test and displayed as mean±s.e.m. *n*≥5 tumours per mouse with *n*≥4 mice per genotype and time point. Scale bar, 50 μm. (**b**) ELISA of VEGFA levels in lung tumour lysates at indicated time points. Data were analysed by Student’s *t*-test and displayed as mean±s.d., *n*≥6 mice per genotype and time point. (**c**) Expression levels of *Pdgfa* in total lungs were measured by quantitative real-time PCR at indicated time points. Values are presented as fold change of relative mRNA expression compared with *Stat3*^*ΔLep/ΔLep*^*:Kras*^*+/+*^ mice. Data were analysed by one-way analysis of variance (ANOVA) with Tukey’s multiple comparison test and are shown as mean± s.d., *n*≥5 animals/genotype. (**d**) Flow cytometric analysis of Cd11b^+^Gr1^+^ granulocytes and Cd11b^+^ F4/80^+^ macrophages in bronchoalveolar lavage (BAL) at 6 and 13 weeks post AdCre. Data were analysed by one-way ANOVA with Tukey’s multiple comparison test and are shown as mean±s.e.m. (**e**) Flow cytometric analysis of myeloid-derived suppressor cell (MDSC) subsets at 6 and 13 weeks post AdCre. Data were analysed by one-way ANOVA with Tukey’s multiple comparison test and are shown as mean± s.e.m. (**f**) Ratio of CD4^+^/CD8^+^ T-cell counts in BAL are shown. Data were analysed by Kruskal–Wallis test with Dunn’s multiple comparison testing and are shown as mean±s.e.m. Data displayed in **d**–**f** are *n*≥6 mice per genotype and time point, 13-week group represents two independent experiments. (**g**) Flow cytometric analysis of Cd11b^+^Gr1^+^ and Cd11b^+^ F4/80^+^ cells of A549 sh*Control* versus A549-sh*STAT3* xenograft tumours. Data were analysed by Student’s *t*-test and are displayed as mean±s.e.m. (*n*≥8 tumours; ≥4 mice per group). (**h**) IHC analysis and representative pictures of CD31^+^ counts per xenograft tumour area (mm^2^). Scale bar, 100 μm (*n*=8 tumours/≥4 mice). Data were analysed by Student’s *t*-test and are shown as mean±s.e.m. For all graphs: *P<0.05; **P<0.01; ***P<0.001.

Detailed histopathological analysis revealed that *Stat3*-deficient murine tumours were better vascularized at all investigated time points, which was corroborated by increased CD31^+^ and von Willebrand Factor staining of vessel walls (Fig. 2a). Tumour angiogenesis is mainly driven by vascular endothelial growth factor alpha (VEGFA) and platelet-derived growth factor alpha (PDGFA) in response to certain stimuli derived from the tumour cells directly or from the surrounding microenvironment^28^. ELISA revealed significantly increased VEGFA levels in lungs from *Stat3*^*ΔLep/ΔLep*^: *Kras*^*G12D/+*^ mice compared with their littermate controls at 6 weeks, whereas at 13 weeks post AdCre infection no difference could be observed (Fig. 2b). However, at that later time point, *Pdgfa* expression was significantly elevated in *Stat3*^*ΔLep/ΔLep*^:*Kras*^*G12D/+*^ mice, which might contribute to enhanced tumour vascularization indirectly through the recruitment of angiogenic stroma cells^29^,^30^ (Fig. 2c).

It has been shown that tumour cell-specific *Stat3* blockade leads to changes in the tumour microenvironment and increased immune cell infiltration^31^. Qualitative IHC analysis of the tumour microenvironment revealed that several cell types are present within the lung tumours (NK-cells, T cells, dendritic cells, fibroblasts and macrophages. Supplementary Fig. 3a). As macrophages and granulocytes contribute to tumour angiogenesis^32^, we performed quantitative assessment of these cell types via flow cytometry analysis of bronchoalveolar lavage (BAL) and observed significantly more CD11b^+^Gr1^+^ granulocytes in *Stat3*^*ΔLep/ΔLep*^:*Kras*^*G12D/+*^ lungs compared with controls at 6 and 13 weeks post AdCre-induction (Fig. 2d). Furthermore, infiltrating CD11b^+^F4/80^+^ macrophages were significantly elevated within tumours of *Stat3*^*ΔLep/ΔLep*^:*Kras*^*G12D/+*^ mice at 13 weeks post AdCre in BAL, which was confirmed by IHC (Fig. 2d and Supplementary Fig. 3b). Further analysis of myeloid-derived suppressor cells (MDSCs), which are known to play an important role in suppressing the antitumour immune response^33^, showed that MDSCs from *Stat3*-deficient mice were more differentiated towards a monocytic phenotype (CD11b^+^Ly6C^high^Ly6G^low^, M-MDSCs) than to a granulocytic phenotype (G-MDSCs, CD11b^+^Ly6C^low^Ly6G^high^) at later stages of tumour development (13 weeks; Fig. 2e). M-MDSCs have been postulated to be more potent in suppressing T-cell responses^33^. In line with this, we found that *Stat3*^*ΔLep/ΔLep*^: *Kras*^*G12D/+*^ animals showed an increased CD4^+^/CD8^+^ ratio compared with control mice at the latest time point (13 weeks), indicating a suppression of the cytotoxic CD8^+^ T cells response within the tumours (Fig. 2f).

We further investigated tumour microenvironment and angiogenesis within our patient cohort. Correlations between CD31 or VEGF and P-STAT3 or STAT3 did not reveal any significant differences between *KRAS* wild-type and mutant cases (Supplementary Fig. 3c,d). Qualitative assessment of infiltrating immune cells revealed that only 3% of tumours were infiltrated with macrophages and we did not observe any differences between both genotypes (Supplementary Fig. 3e). However, the number of tumours scoring positive for infiltrating granulocytes was higher in *KRAS* mutant compared with *KRAS* wild-type tumours (18% within the *KRAS* mutant versus 10% within *KRAS* wild-type cohort); in addition, less cases of *KRAS* mutant tumours scored positive for lymphocyte infiltration compared with *KRAS* wild-type tumours (31% within the *KRAS* mutant versus 48% in *KRAS* wild-type cohort; Supplementary Fig. 3e).

In order to substantiate the findings of our mouse model, we analysed the human AC xenograft samples and found that tumours lacking *STAT3* showed significantly increased numbers of infiltrating CD11b^+^Gr1^+^ and CD11b^+^ F4/80^+^ cells (Fig. 2g) and were significantly more vascularized (Fig. 2h), thus confirming our observations in the murine model. Together, these results implicate that *STAT3* ablation results in a considerably different tumour microenvironment composed of more myeloid cells, increased CD4^+^/CD8^+^ lymphocyte ratio and enhanced angiogenesis in our murine as well as in the human AC xenograft models.

### STAT3 regulates chemoattractive CXCL1 expression

**Figure 3:**
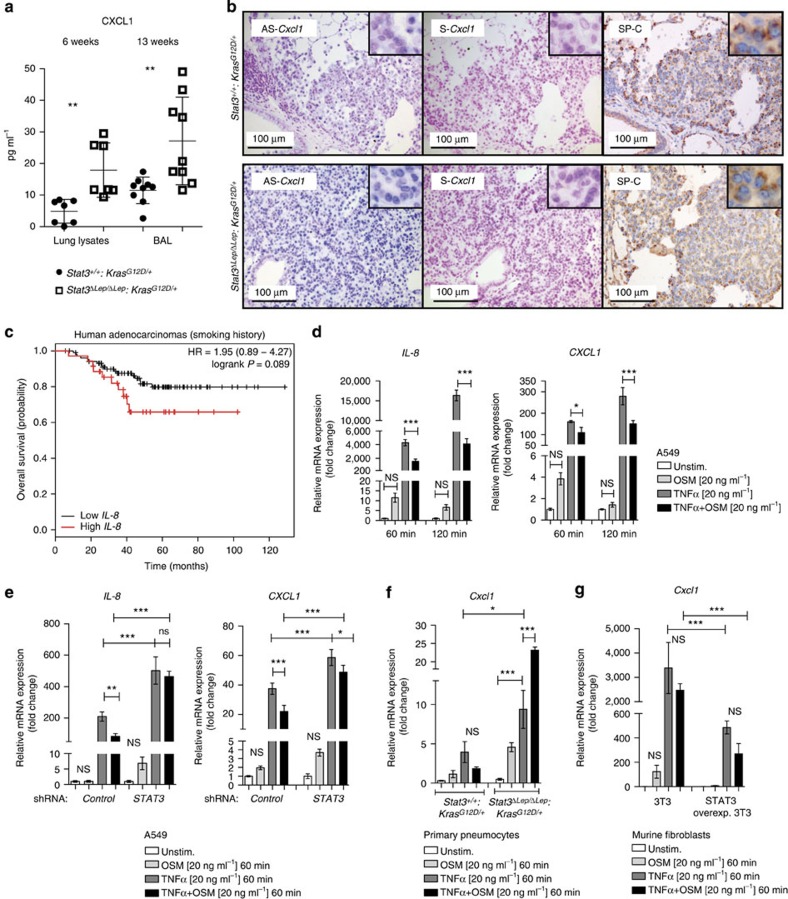
STAT3 regulates chemoattractive CXCL1 expression. (**a**) ELISA of CXCL1 levels in lung lysates and BAL at 6 and 13 weeks post AdCre. Data were analysed by Student’s *t*-test and shown as mean±s.d., *n*≥7 mice per genotype and time point. (**b**) Representative *in situ* hybridization with probes specific for murine *Cxcl1* (AS) in lung tumours at 13 weeks post AdCre. Sense probes (S) were used as negative control and surfactant protein C (SP-C) as lung epithelial maker. Scale bar, 100 μm. (**c**) Kaplan–Meier plot showing overall survival of lung adenocarcinoma patient samples harbouring smoking history with high or low *IL-8* mRNA expression (expression values of upper quartile, log-rank test) *n*≥35–104 per group. (**d**) Human A549 lung cancer cell line (*KRAS*^*G12S*^ mutated) was stimulated with designated cytokines for indicated time points. *IL-8* or *CXCL1* expression was analysed via qRT–PCR. Data were analysed by one-way ANOVA with Tukey’s multiple comparison test and shown as mean±s.d. (**e**) A549 cells transducted with lentiviral vectors expressing nonspecific scrambled small hairpin (sh)RNA or shRNA against *STAT3* were stimulated with indicated cytokines for 60 min. Data were analysed by one-way ANOVA with Tukey’s multiple comparison test and shown as mean±s.d. (**f**) Primary pneumocytes were isolated and infected with AdCre. 120 h post infection cells were stimulated with indicated cytokines for 60 min. *Cxcl1* expression was analysed by qRT–PCR. Data are shown as mean±s.e.m. (**g**) Murine 3T3 fibroblasts and STAT3 overexpressing 3T3 fibroblasts were stimulated as in **f**. *Cxcl1* expression was analysed by qRT–PCR. Data were analysed by one-way ANOVA with Tukey’s multiple comparison test and shown as mean± s.d. Values in **d**–**g** are presented as fold change of relative mRNA expression compared with each unstimulated individual cell line. At least two independent experiments with three individual plates per stimulation were performed. For all graphs: *P<0.05; **P<0.01; ***P<0.001.

To test if the increased recruitment of myeloid cells is a result of changes within the local cytokine and growth factor milieu in *Stat3*^*ΔLep/ΔLep*^:*Kras*^*G12D/+*^ tumours, we performed ELISA of several key cytokines, which may modulate the infiltration of myeloid cells into tumours. IL-2, IL-12p40, IL-4 and IL-17 were not detectable and we did not find differences in IL-5, interferon-γ, IL-10, IL-1α, EGF, OSM expression between the two genotypes (Supplementary Figs 4a and 1b). Granulocyte–macrophage colony-stimulating factor and IL-6 expression were upregulated in *Stat3*-deficient tumours only at the latest time point (Supplementary Figs 4a and 1b). Interestingly, CXCL1 was the only cytokine, which showed a significant increase in lungs from *Stat3*^*ΔLep/ΔLep*^:*Kras*^*G12D/+*^ mice compared with their control littermates at all time points (Fig. 3a). *Cxcl1* (murine orthologue to human *CXCL1* and *IL-8* (ref. 34)) is a well-known and potent chemo-attractant for macrophages and granulocytes and can induce angiogenesis^35^,^36^. *In situ* hybridization not only corroborated the increased expression of *Cxcl1* in *Stat3*-deficient tumours but also identified the tumour cells as the main cellular origin of *Cxcl1* as confirmed by staining for the alveolar type 2 cell-specific marker SP-C (surfactant protein C) in serial sections (Fig. 3b and Supplementary Fig. 4b). Together, these data suggest that STAT3 negatively regulates the angiogenic and pro-inflammatory cytokine CXCL1 in the murine model.

Next, we analysed the patient cohorts for *IL-8* expression, the human orthologue of *Cxcl1* (ref. 34). Interestingly, STAT3 and IL-8 expression tended to inversely correlate in *KRAS* mutant samples, whereas *KRAS* wild-type samples showed a trend towards a positive correlation between STAT3 and IL-8 expression (Supplementary Fig. 4c). Furthermore, high *IL-8* expression levels have been associated with poor prognosis in smoking patients^37^, a finding which we could confirm within the second patient cohort used in this study (Fig. 3c)^26^.

We next performed various cytokine stimulation experiments *in vitro* to corroborate the *Stat3*-mediated repression of *Cxcl1* observed in the murine model. First, we confirmed that the human *KRAS* mutant AC cell line A549 activates STAT3 in response to cytokines such as OSM, IL-6 and EGF found to be expressed in our murine lung tumours *in vivo* (Supplementary Figs 1b and 4d). We used tumour necrosis factor-α (TNF α) to stimulate NF-κB induced *IL-8* and *CXCL1* expression in A549 cells and analysed the effect of co-stimulating STAT3 by OSM or IL-6. As expected, TNF α stimulation induced a marked *IL-8* and *CXCL1* expression, which was significantly inhibited by co-treatment with OSM (Fig. 3d). Interestingly, OSM treatment alone also led to a very small and transient increase of *IL-8* and *CXCL1*. Similar results were obtained with IL-6 co-stimulation (Supplementary Fig. 4e). Importantly, small hairpin-mediated knockdown of *STAT3* confirmed that the observed effects on *CXCL1* and *IL-8* repression were at least in part mediated by STAT3 (Fig. 3e and Supplementary Fig. 4f).

Next, we isolated primary mouse alveolar type 2 pneumocytes from the lungs of *Stat3*^flox/flox^:*Kras*^LSL-G12D/+^ and *Stat3*^+/+^:*Kras*^LSL-G12D/+^ animals (Supplementary Fig. 4g). Complete deletion of STAT3 was observed 120 h post AdCre infection *in vitro* (Supplementary Fig. 4h). In these primary cells, TNF α treatment again triggered *Cxcl1* expression albeit less pronounced compared with A549 cells (Fig. 3f). Importantly, *Stat3*-deficient *Kras*^G12D/+^-mutant pneumocytes expressed significantly increased levels of *Cxcl1* upon TNF α stimulation compared with syngeneic *Stat3*-proficient cells. Upon co-stimulation with OSM, *Stat3*-deficient pneumocytes not only failed to repress *Cxcl1* expression but even showed vastly increased *Cxcl1* expression levels (Fig. 3f). Conversely, experiments using murine 3T3 fibroblasts overexpressing STAT3 (ref. 10) showed that STAT3 is not only required but is sufficient to repress *Cxcl1* expression (Fig. 3g). These results suggest that STAT3 represses *Cxcl1* expression *in vivo* and *in vitro*.

### CXCL1 inhibiton reverts oncogenic effects of STAT3 ablation

**Figure 4:**
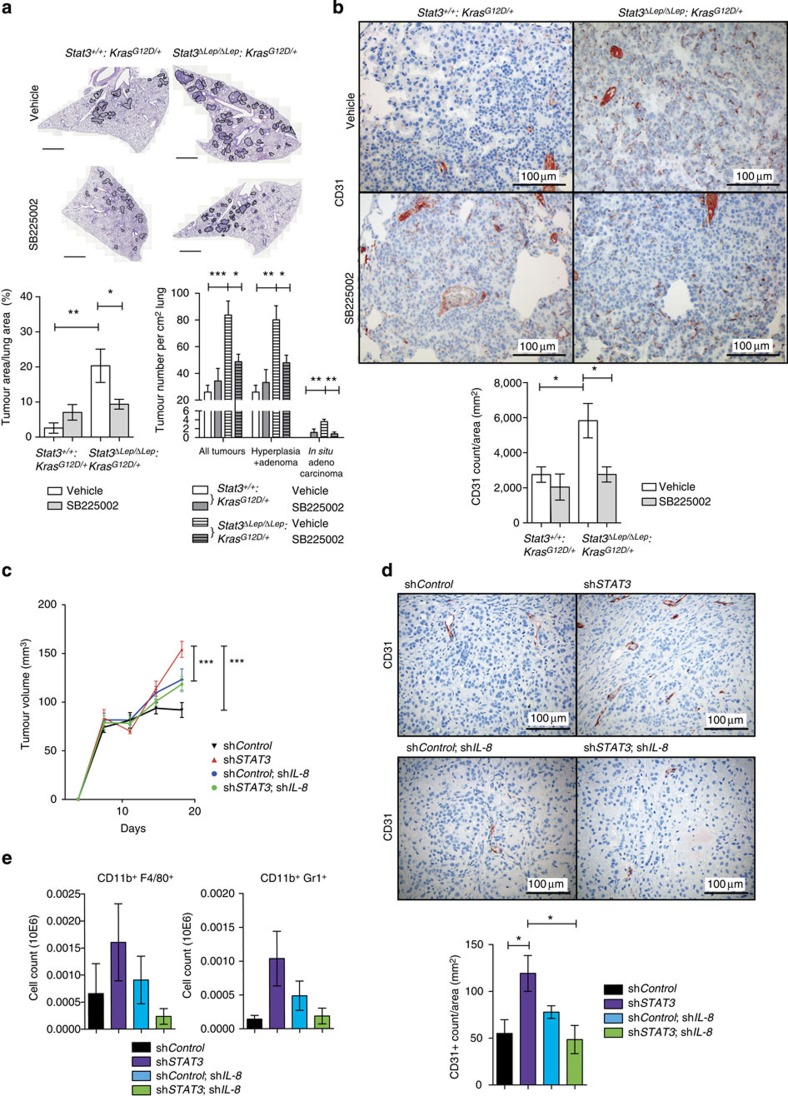
CXCL1 inhibiton reverts oncogenic effects of STAT3 ablation. (**a**) Mice were treated with the CXCR2 antagonist SB225002 or vehicle control starting 1 week after tumour induction and euthanized after 5 weeks (treatment 1 shown in Supplementary Fig. 5b). Tumour area/lung area was quantified within each group and at least two sections of each lung were stained with haematoxylin and eosin and analysed in a blinded manner. Tumour grading is shown in the right panel (*n*=4–7 mice per genotype). Data in both panels were analysed by one-way ANOVA with Tukey’s multiple comparison test and shown as mean±s.e.m. Scale bar, 2 mm. (**b**) Tumour vascularization was quantified by CD31^+^ counts per tumour area (mm^2^). At least 3 tumours per mouse were analysed with *n*=4–7 mice per genotype and treatment. Data were analysed by one-way ANOVA with Tukey’s multiple comparison test and shown as mean±s.e.m. Scale bar, 100 μm. (**c**) Short hairpin-mediated knockdown of IL-8 (sh*IL-8*) in either sh*Control* or sh*STAT3* A549 NSCLC cells were performed and 2 × 10^6^ cells were injected in both flanks of male nude mice (*n*=5 per group). Xenograft tumour growth was determined at indicated time points. Data were analysed by Two-way ANOVA with Bonferroni multiple comparison test and shown as mean±s.e.m. (**d**) IHC analysis of CD31^+^ counts per tumour area (mm^2^) showed reduced vascularization of A549-sh*STAT3*;sh*IL-8* xenograft tumours compared with controls. Data were analysed by one-way ANOVA with Tukey’s multiple comparison test and shown as mean±s.e.m. Scale bar, 100 μm. (**e**) Flow cytometric analysis of Cd11b^+^Gr1^+^ granulocytes and Cd11b^+^ F4/80^+^ macrophages displayed reduced myeloid infiltration in A549-sh*STAT3*;sh*IL-8* xenograft tumours compared with controls (*n*≥8 tumours; 5=mice per group). At least 6 tumours per mouse were analysed with *n*≥7 mice per genotype and treatment. Data were analysed by Kruskal–Wallis test with Dunn’s multiple comparison testing and shown as mean±s.e.m. For all graphs: *P<0.05; **P<0.01; ***P<0.001.

Next, we tested whether CXCL1 is responsible for the increased tumour growth supported by vascularization and infiltration. We used the SB225002 compound to inhibit CXCL1’s cognate receptor CXCR2, which we found to be expressed on epithelial, endothelial and myeloid cells within the lung tumours (Supplementary Fig. 5a)^36^,^38^. CXCR2 antagonist treatment was conducted in two ways: first, we treated the mice 1 week after AdCre tumour induction (Supplementary Fig. 5b). After 5 weeks of continuous CXCR2 blockade, we could not only observe a marked reduction in tumour vascularization but importantly tumour incidence and overall tumour burden were reduced, suggesting that CXCL1 is crucial for tumour development at early stages in *Stat3*^*ΔLep/ΔLep*^: *Kras*^*G12D/+*^ mice (Fig. 4a,b). Second, we tried to mimic a therapeutic setting and treated tumour-bearing mice (Supplementary Fig. 5b). After 7 weeks of treatment, we could observe a significant reduction of vascularization and of infiltrating F4/80^+^ macrophages in the tumours of *Stat3*^*ΔLep/ΔLep*^: *Kras*^*G12D/+*^ mice (Supplementary Fig. 5c), but not in overall tumour burden (Supplementary Fig. 5d). To further confirm these findings in a human cell line, we performed a small hairpin-mediated double knockdown of *IL-8* and *STAT3* in A549 cells (Supplementary Fig. 5e). Indeed, enhanced tumour growth of sh*STAT3* cells was significantly reduced upon knockdown of *IL-8* (Fig. 4c). Furthermore, vascularization as well as macrophage and granulocyte infiltration was reduced in the *IL-8*/*STAT3* double knockdown tumours compared with *STAT3* knockdown tumours (Fig. 4d,e). Interestingly, knockdown of *IL-8* alone showed an increased tumour growth in nude mice compared with sh*Control* cells for unknown reasons (Fig. 4c). Taken together, these data suggest that loss of STAT3 in the tumour cells results in increased CXCL1/IL-8 expression triggering infiltration of myeloid cells as well as augmented vascularization, and identifies CXCL1/IL-8 as an essential mediator for the accelerated development and progression of *Stat3*-deficient tumours.

### STAT3 retains p65 in the cytoplasm to reduce NF-κB activity

**Figure 5:**
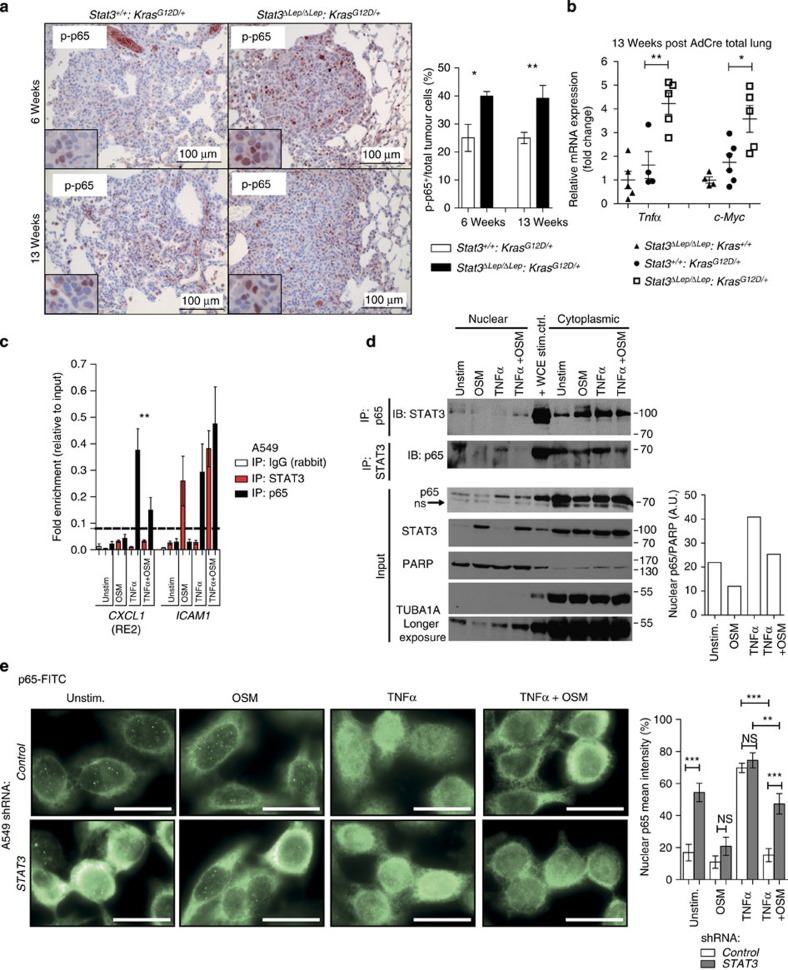
STAT3 retains p65 in the cytoplasm to reduce NF-κB activity. (**a**) NF-κB subunit p65 activation status (p-p65) was analysed by IHC. *n*≥5 tumour sections per mouse per genotype and time point. Data were analysed by Student’s *t*-test and are shown as mean±s.d. Scale bar, 100 μm. (**b**) qRT–PCR of NF-κB subunit p65 target genes (*Tnfα*, *c-Myc*) from total lungs at 13 weeks post AdCre. *n*≥4 mice per genotype. Data were analysed by one-way ANOVA with Tukey’s multiple comparison test and are displayed as mean±s.e.m. (**c**) Chromatin immunoprecipitation (ChIP) of NF-κB subunit p65 or STAT3 binding on human *CXCL1* (responsive element 2, RE2). A549 cells were stimulated with indicated cytokines for 10 min. Binding of STAT3 and p65 on *ICAM1* promoter served as positive control. *ACTB* element was chosen as negative binding region, indicated by the horizontal line. Values are presented as fold enrichment relative to chromatin input. Data of two independent experiments are shown. Data were analysed by one-way ANOVA with Tukey’s multiple comparison test and shown as mean±s.e.m. (**d**) A549 cells were stimulated as in **c**. Immunoprecipitation (IP) of nuclear and cytoplasmic fractions was performed with antibodies against p65 or STAT3 and subjected to STAT3 and p65 immunoblot analysis, respectively. IP inputs were subjected to STAT3 and p65 (arrow indicates unspecific binding). PARP (nuclear) and TUBA1A (cytoplasmic) were used to determine purity of input. Densitometric quantification of bands was performed (AU, arbitrary units). (**e**) A549 cells transducted with scrambled shRNA or shRNA against *STAT3* were stimulated as in **c** and stained with antibodies for NF-κB subunit p65 (FITC). *n*≥6 regions of interest were quantified. White scale bar, 20 μm. Data were analysed by one-way ANOVA with Tukey’s multiple comparison test and shown as mean± s.e.m. For all graphs: *P<0.05; **P<0.01; ***P<0.001.

Having demonstrated the importance of STAT3-mediated repression of *CXCL1* in tumour cells *in vitro* and *in vivo*, we set out to mechanistically interrogate this pathway further. As NF-κB subunit p65 is an important regulator of *CXCL1* expression, we first examined the p65 activation^39^,^40^. Increased levels of activated p65 were observed, which was mirrored by increased expression of *NFκB-p65* target genes such as *Tnfα*, *c-Myc, Il-6* and *Cxcl1* in *Stat3*-deficient lungs, whereas *Bcl2, Bcl2l1, Ccnd1* and *Ccl5* were comparably expressed (Figs 3a,5a,b; Supplementary Figs 1b and 6b)^41^. These data demonstrate that *Stat3*-deficient tumours show increased *NF-κB* activity.

We examined the association of *NF-κB* and *STAT3* expression in our patient sample archive. We found a significant positive correlation of *NF-κB* and *STAT3* expression in *KRAS* wild-type samples. On the contrary, we found a trend towards an inverse correlation between *NF-κB* and *STAT3* expression in *KRAS* mutant samples (Supplementary Fig. 6c).

As STAT3 has been shown to compete with NF-κB on various promoters^42^,^43^, we further analysed the crosstalk of STAT3 and NF-κB binding on the human CXCL1 and the mouse *Cxcl1* promoter. Within the mouse *Cxcl1* promoter, we found several putative STAT3-binding sites—two of them overlapping with putative NF-κB-binding sites and conserved in humans (hereafter termed responsive element, *RE1* and *2*; Supplementary Fig. 6d). However, electrophoretic mobility shift assays of all putative binding sites (Supplementary Fig. 6e) as well as *in vivo* chromatin immunoprecipitation experiments could not reveal strong endogenous binding of STAT3 on *RE1* or *RE2* (Supplementary Fig. 6f). We next tested the human A549 cell line focusing on the *RE2* site (Fig. 5c). Upon TNF α treatment, we could detect efficient recruitment of p65 to the CXCL1 promoter and co-stimulation of STAT3 signalling by OSM treatment indeed markedly reduced p65 recruitment. However, we failed to detect direct STAT3 binding at the CXCL1 promoter site (Fig. 5c), indicating that STAT3 regulates p65-induced CXCL1 expression by means other than competing for promoter-binding sites.

As STAT3 and the NF-κB subunit p65 have been reported to interact at the protein level^43^,^44^,^45^, we next tested if STAT3 may bind and control subcellular localization of p65. Co-immunoprecipitation experiments in A549 cells showed that the majority of STAT3 and NF-κB–p65 interact in the cytoplasm under basal conditions as well as upon stimulation with OSM, TNF α or a combination thereof (Fig. 5d). We next quantified nuclear p65 levels normalized to poly (ADP-ribose) polymerase family, member 1 (PARP). Upon OSM stimulation, a reduction of nuclear p65 compared with unstimulated conditions was observed. Increased levels of nuclear p65 were obtained upon TNF α stimulation, which was reduced to basal levels upon co-stimulation with OSM (Fig. 5d). To further confirm this, we analysed the subcellular localization of p65 using immunofluorescence imaging of STAT3-proficient A549 cells and compared it to the STAT3 knockdown cell line (Fig. 5e). Under basal conditions, A549-sh*STAT3* cells already showed increased levels of nuclear p65 compared with controls. Interestingly, we observed a reduction of nuclear p65 in OSM-stimulated A549-sh*STAT3* cells compared with unstimulated controls, indicating that additional OSM-induced effectors other than STAT3 are also regulating the cytoplasmic-nuclear trafficking of p65. As expected, both cell lines showed increased levels of nuclear p65 upon TNF α stimulation. Importantly, co-stimulation with OSM resulted in a significant reduction of TNF α-induced nuclear p65 accumulation in A549 scrambled control cells but not in sh*STAT3*-infected cells (Fig. 5e). In summary, our results suggest that STAT3 represses NF-κB-dependent *CXCL1* expression by sequestering NF-κB in the cytoplasm and mechanistically delineate a novel tumour-suppressive pathway governed by the STAT3–NF-κB–CXCL1 axis.

## Discussion

Personalized treatment decisions based on the genetics of the individual tumour will be paramount to combat malignancies in the near future. STAT3 has been implicated in several malignancies^5^,^7^,^13^,^14^,^46^ and therefore, clinical studies are currently evaluating the efficacy of STAT3 inhibition in various kinds of cancer^46^. However, although STAT3 traditionally has been described as an oncogene^7^,^9^, recent reports have shown that STAT3 can also behave as a tumour suppressor in the very same organ systems^9^,^11^,^12^,^42^. In addition, other reports validated high STAT3 expression as a potential marker for good prognosis in human colorectal carcinoma and breast cancer^47^,^48^. Therefore, the idea emerged that STAT3 function is context-dependent either with regards to the oncogenic driver mutation, cancer type or the specific tumour microenvironment.

Specifically in lung cancer, STAT3 has been shown to function as one of the main downstream transcription factors in *EGFR* mutant ACs^13^ and to act as an oncogene in a chemically induced mouse model of lung cancer^49^. In addition, several studies of human lung cancer specimens have shown the activation of STAT3 in those tumours^13^,^14^,^16^. These findings made STAT3 an attractive drug target to treat NSCLC patients. We hypothesized that lung AC patients, especially those with activating *KRAS* mutations, might also benefit from STAT3 inhibition. As *KRAS* is still considered as an undruggable target and is responsible for 30% of all Caucasian AC cases, we investigated whether STAT3 inhibition has a potential survival benefit in a preclinical lung cancer mouse model and in a human cell line xenograft model. However, our results clearly show that STAT3 behaves as a haploinsufficient tumour suppressor during *Kras*^*G12D*^-induced lung tumorigenesis. Mice lacking *Stat3* signalling specifically within *Kras*^*G12D*^-mutant tumour cells not only display increased tumour initiation and tumour cell proliferation but also accelerated malignant progression and ultimately markedly reduced survival compared to mice with intact *Stat3* signalling. We could confirm these observations in human lung AC cell-derived xenografts. Of note, others have shown that reducing STAT3 activity by using STAT3 inhibitors or STAT3 decoy oligonucleotides suppressed tumour growth in xenograft-derived NSCLC cell lines^18^,^50^,^51^. However, by using a powerful short hairpin RNA (shRNA)-mediated loss-of-function approach, we found that STAT3 suppresses tumorigenesis in A549 cell-derived xenografts, thus supporting our findings in the murine model. Despite the fact that *Stat3*-deficient tumours had a growth advantage, they did not progress to become more invasive than *Stat3*-proficient tumours or metastatic. Tumour invasion and metastasis are a multistage development in which, in addition to neo-angiogenesis, malignant tumour cells have to undergo EMT, detach from the primary tumour, migrate and pass mechanical barriers. This is a complex process driven by multiple somatic aberrations. Thus, most likely, *Stat3*-deficient tumours need additional hits to become invasive and to metastasize.

Interestingly, the observed survival disadvantage is more pronounced in male mice than in female mice reminiscent of observations made in the clinic: although women have a higher incidence rate in developing lung cancer, they have a better survival outcome compared with men^52^. Further on, STAT3 already has been linked to gender-specific diseases in liver and lung cancer^53^,^54^. Detailed analysis of this gender-specific difference associated with STAT3 will be of high interest. One of the main pro-inflammatory and neo-angiogenesis promoting cytokines responsible for our observations in *Stat3*-deficient tumours is *Cxcl1*. *Cxcl1*, the murine orthologue to human *IL-8* and *CXCL1*, is known to be RAS dependent^40^, to directly attract myeloid cells and to stimulate endothelial cells to promote angiogenesis^35^,^36^. Clinically, IL-8 expression is associated with a tendency for poor prognosis in human lung cancer^37^ and expression of CXCR2 also correlates with smoking and poor prognosis^55^. In agreement with this, we could confirm that *IL-8* expression tends to correlate with poor survival in smoking patients (who frequently acquire *KRAS* mutations) within the publically available AC patient cohort used in this study. Interestingly, we could also show that low STAT3 expression has a tendency to correlate with increased *IL-8* mRNA expression in *KRAS* mutant patient samples.

To investigate whether CXCL1 is the central angiogenesis and tumour-promoting factor in our model and a potential therapeutic target, we treated the mice with SB225002, a small-molecule antagonist of CXCR2. Treating *Stat3*-deficient mice at tumour initiation resulted in a reduction of tumour growth as well as a decrease in vascularization compared with vehicle-treated control mice. This result corroborates that CXCL1 signalling indeed accelerates tumour growth at early stages. In addition, we cannot rule out that CXCL1 also acts as an autocrine tumour-promoting factor in lung cancer cells^56^,^57^,^58^. We also tried to validate CXCL1 as a therapeutic target by blocking CXCL1 signalling in established tumours. By using this approach, we could show that tumour vascularization and infiltration of macrophages were reduced at the end stage of the treatment; however, we did not observe any effect on tumour growth. This result indicates that other effectors in *Stat3*-deficient tumours, like elevated NF-κB activity and/or increased *c-Myc* expression, may contribute to tumour proliferation at late stages. Nevertheless, although late-stage *Stat3*-deficient tumours failed to respond to an anti-CXCL1 monotherapy, the use of CXCL1 blockers combined with standard chemotherapy or as maintenance therapy could be a reasonable alternative to treat these tumours and it deserves further investigation. To substantiate these findings, we inhibited IL-8 signalling in *STAT3* knockdown human lung AC cells. Short hairpin-mediated *IL-8*/*STAT3* double knockdown resulted in a reduction in tumour growth, tumour vascularization and macrophage infiltration compared with *STAT3* single knockdown within the human A549 xenograft model. These results confirm the central role of CXCL1/IL-8 signalling in mutated *KRAS* STAT3-deficient murine and human xenograft tumours.

Mechanistically, we show that STAT3 signalling can repress NF-κB-driven *CXCL1/IL-8* expression not only in primary mouse pneumocytes but also in human lung AC cells. Furthermore, we show that tumours lacking *Stat3* showed increased nuclear p-p65 concomitant with increased expression of CXCL1 and other typical NF-κB target genes, such as *Tnfα, c-Myc* and *Il-6*, which was also shown by others in A549 cells as well as in glioblastoma cells upon STAT3 blockade^59^,^60^. Although STAT3 as well as other STAT family members has been shown to compete with NF-κB on various promoter regions^43^,^61^,^62^, we could not detect direct STAT3 binding to the *CXCL1* promoter. We found that activation of STAT3 results in binding to and retention of NF-κB in the cytoplasm, thereby reducing NF-κB transcriptional activity. In line with these results, *Stat3* deficiency in dendritic cells^63^ and splenocytes as well as knockdown or inhibition of STAT3 within melanoma or prostate cancer cell lines^44^ causes increased phosphorylation of IκBα, indicating that STAT3 can also impinge on NF-κB activity further upstream in the inhibitory complex formation of IκBα and NF-κB.

Interestingly, mucinous lung AC patients with *KRAS* mutations not only show reduced STAT3 expression levels but also reduced STAT3 activation levels compared with smoking patients without *KRAS* mutations and to *EGFR* mutant cases. Further analysis revealed that more tumours with high granulocyte and low lymphocyte infiltration were found within *KRAS* mutant patient samples. In addition, low STAT3 expression tends to correlate with increased NF-κB and *IL-8* expression. Nevertheless, increased patient numbers are needed to substantiate these findings. Furthermore, in lung AC patients with a known smoking history, a patient subgroup associated with *KRAS* mutations, we found a significant correlation between lower STAT3 expression and poor survival. In line, those tumours with advanced stage (grade III) had significantly less *STAT3* expression, indicating that *STAT3* downregulation seems to be beneficial for tumour progression. Furthermore, two additional independent data sets confirmed that *STAT3* expression was significantly downregulated in high-risk patients with subsequent poor survival outcome in lung AC patients. GSEA further showed that our murine *Stat3*-deficient *Kras*^*G12D*^ tumours strongly correlate with a gene expression signature associated with human *KRAS* mutant lung tumours, and importantly, also with a poor prognosis-gene signature—more than *Stat3*-proficient *Kras*^*G12D*^ tumours. These data suggest that our observations in the *Stat3*-deficient *Kras*^*G12D*^ mouse model are actually reflecting the human situation where tumour grade and poor prognosis correlate with low STAT3 expression in lung AC patients.

Based on *in vitro* studies and xenograft models, STAT3 is considered to play a tumour-promoting role in *EGFR* mutant NSCLC^13^,^14^,^16^,^18^, therefore, inhibition of STAT3 is considered as therapeutic intervention in lung ACs^46^. We have demonstrated that STAT3 has a tumour-suppressor function in murine *Kras* mutant lung cancer as well as in human *KRAS* mutant xenografts. Although further clinical investigations will be required, our data suggest that those lung AC patients harbouring *KRAS* mutations or a known smoking history (as a surrogate marker for *KRAS* mutations), may not respond to STAT3 inhibitors but may likely suffer severe adverse effects. This highlights the importance of stratifying patients according to their driver mutation for further therapeutic interventions. In addition, our study also proposes a re-evaluation of STAT3 inhibitors^46^ as therapeutic strategies for any inflammatory or fibrotic disease or cancer, as inhibition of STAT3 *in vivo* might bear the risk of triggering malignant transformation of incipient pre-malignant cells harbouring *KRAS* mutations within the lung.

## Methods

### Tumour induction and inhibitor administration in mice

Mice carrying the *Stat3*^*floxed*^ allele^20^ were crossed with the *Kras*^*LSLG12D/+*^ knock-in mice^19^ to generate *Stat3*^*flox/flox*^*:Kras*^*G12D/+*^ mice maintained on a C57BL6/N background. In all experiments described, littermates were used as controls. All mice were bred and maintained according to an ethical animal licence protocol complying with the current Austrian law. Induction of lung tumours via intranasal AdCre inhalation was performed with 2.5 × 10^7^ plaque-forming unit as described^64^. *Stat3*^*ΔLep/ΔLep*^*:Kras*^*G12D/+*^ and *Stat3*^*+/+*^*:Kras*^*G12D/+*^ mice were treated with SB225002 (Tocris#2725) as following: 1 week or 6 weeks post Adenoviral Cre infection, mice were injected either with vehicle or SB225002 (dissolved in PBS+0.25% Tween-20) with doses of 0.5 mg kg^−1^ bodyweight intraperitoneally 5 days per week for 5 or 7 weeks, respectively. Treatment was performed according to an animal licence protocol approved by the Bundesministerium für Wissenschaft und Forschung (BMWF-66.009/0252-II/3b/2013).

### Xenograft experiments

A total of 2 × 10^6^ cells were mixed 1:2 with Matrigel (Corning#356231) and injected into left and right flank of Hsd:Athymic Nude-Foxn1^nu^ male mice (3–4 weeks age, Harlan). Tumour volumes were evaluated twice a week by measuring two perpendicular diameters with calipers. Tumour volume was calculated using the following equation: (width*width*length)/2. Treatment was performed according to an animal licence protocol approved by the Bundesministerium für Wissenschaft und Forschung (BMWF-66.009/0280-II/3b/2012).

### Human data

A tissue microarray was produced for mucinous lung ACs. Out of 346 human lung AC cases, 74 were selected that harboured a known smoking history and showed a mucinous differentiation. In comparison to non-mucinous ACs, mucinous subtypes harbour a *KRAS* mutation in 56% of cases (versus 25–30% in non-mucinous AC), thus increasing the probability to identify a significant amount of mutated cases (P.H., unpublished data). Human tissue samples were analysed with prior approval by the institutional ethical committee (24–135 ex 11/12). Gene expression data and overall survival information were analysed as described^26^. In brief, the GEO, EGA and TCGA repositories were searched for suitable databases containing raw microarray and survival data as well as clinical characteristics including smoking history for lung AC samples. Two GEO data sets (GSE29013 and GSE31210) fulfilled these criteria. For all samples in these data sets, normalized gene expression was recomputed using MAS5. Then, to analyse the prognostic value of *STAT3* or *IL-8*, the lung AC patient samples with or without smoking history were split into two groups according to lower/upper quartile expression of *STAT3* or *IL-8*, respectively. The used probe set for *STAT3* was 208991_at and for *IL-8* it was 202859_x_at. The two patient cohorts were analysed by Kaplan–Meier survival plot and the hazard ratio with 95% confidence intervals and log-rank *P* value were calculated. The data sets used to determine the prognostic value of *STAT3* in the lung AC patients^27^ are taken from GSE30219 and based upon data generated by the TCGA Research Network: http://cancergenome.nih.gov/.

### Histology

For histological analysis of lung tumours, 2.5 μm sections from at least two different central planes of the lungs were cut, stained with haematoxylin and eosin and scanned with TissueFaxs software (TissueGnostics GmbH). Quantification of tumour area, tumour number and tumour grade was done with HistoQuest software (TissueGnostics GmbH) and visually controlled by two independent pathologists (R.H.Z. and H.P.) in a blind manner. Tumour grade was classified as following: hyperplasia includes diffuse hyperplasia of pneumocytes type II and adenomas. The first is a diffuse proliferation, whereas adenomas are circumscribed. Adenomas can be composed of transformed pneumocytes type II but also can contain some Clara cells. Both lesions represent non-malignant proliferations, have a compact cell mass, alveolar septae are still intact and the nuclear/cytoplasmic ratio is still regular. *In situ* ACs grow along preexisting alveolar septae, have prominent nucleoli and an increased nuclear/cytoplasmic ratio, but still low mitotic counts per high-power field. Sometimes this proliferation appears pseudosolid, because of alveolar collapse or increased cell size obscuring the alveolar lumen. Into this category we also included intrabronchiolar papillary neoplasia as the lesion from which *in situ* ACs develop. Invasive ACs were classified by harbouring desmoplastic stroma formation (confirmed by Movat pentachrome staining: elastic lamina are stained black-brown; collagen III and V are stained green; collagen I is stained yellow; cells are stained dark red; mucins are stained blue.), invasion into the desmoplastic stroma or lymphatic or blood vessels and high mitotic counts per high-power field.

### *In situ* hybridization and IHC

Digoxigenin labelled RNA fragments for *Cxcl1* were used as probes for *in situ* hybridization. Human *in situ* hybridization was performed used the RNAscope 2.0 High Definition assay^65^ according to the manufacturer’s instructions (Advanced Cell Diagnostics, Inc.) to detect human *IL8* transcripts (Probe—Hs-IL8, 310386). As positive and negative control, we used the RNAscope *Hs-POLR2A* (310451) and *dapB* (310043) control probe sets. IHC was performed using antibodies against P-STAT3 (Cell Signaling#9145, 1:100), ser276 p-p65 (Cell Signaling#3037; 1:50), p65 (Santa Cruz#372, 1:200), total STAT3 (N-terminal, BD#610189; 1:200), CD31 (Dianova#DIA-310, 1:50), SP-C (Santa Cruz#13979, 1:100) and F4/80 (AbD Serotech#MCA497G, 1:80), NKp46 (BioLegend#137602; 1:200), Cd11c (Biolegend 117301, 1:200), CD3 (Neomarkers RM9107, 1:100), FSP1 (Millipore 07–2274, 1:600) using standard protocols. At least five different high-power field sections ( × 200 objective) per mouse and per genotype were compared. Quantification for IHC were either done with HistoQuest or for IF staining with TissueQuest (TissueGnostics, GmbH). Image analysis parameters (cell size, cell shape, nuclear size, nuclear shape) were used to discriminate between stroma and tumour cells and were adjusted under the supervision of two board certified pathologist (H.P. and R.H.Z.) to ensure that quantification of P-STAT3 staining in tumour stroma cells versus tumour cells was properly performed.

### Microarray analysis

Total RNA from four tumours/genotype at 13 weeks post AdCre were isolated using the RNAeasy Kit (Qiagen#74104), pooled and hybridized to GeneChip Mouse Gene 1.0 ST array (Affymetrix). GSEA was performed to analyse changes in functional gene sets^66^. Microarray data and description of experimental design are deposited under GEO number GSE52798.

### Cytokine measurement

Cytokine Bead Array (eBioscience #BMS820FF and #BMS86019FF) was done with the BAL of mice at 13 weeks post AdCre. In short, lungs were instilled and flushed two times with 1 ml sterile phosphate-buffered saline each. The cytokine bead array was performed according to manual instructions, measured with FACS Canto II (BD Biosciences) and analysed with FlowCytomix Pro 2.4 (eBioscience). For lung lysates, a piece of frozen lung was homogenized in phosphate-buffered saline and three freeze–thaw cycles were performed to breakup cellular membrane. Afterwards supernatants were used and ELISA was performed according to the manual instructions (R&D #M6000B, #MEG00, #MKC00B, #AF495NA, #BAF495, #495MO025; eBioscience #BMS612). For labelling of infiltrating immune cells, BAL of mice at 6 and 13 weeks post AdCre was used. Single-cell suspensions were labelled overnight at 4 °C with appropriate antibodies in phosphate-buffered saline. The number of macrophages, granulocytes and MDSCs are defined by the total number of cells in BAL multiplied by the percentage of each cell type identified by flow cytometry (FACS Canto II (BD Biosciences)) and analysed by FlowJo (TreeStar, Inc.).

### *In vitro* experiments and isolation of primary pneumocytes

A549 cells were supplied from ATCC and maintained at standard conditions. STAT3α overexpressing 3T3 cells and 3T3 control cells were provided by R.M. and described elsewhere^10^. Stimulation experiments were done with human OSM (#11344022), human IL-6 (#11340060), human TNF α (#11343013), murine IL-6 (#12340063) and murine TNF α (#352392), all from Immunotools. Murine OSM (#495MO025) was purchased from R&D. At least two independent experiments with three individual plates per stimulation were performed. Isolation of primary pneumocytes was performed as described previously^22^. Briefly, lungs were inflated with 3 ml Dispase II (BD#354235), digested with 0.01% DNase1 (SIGMA#D5319) and filtered through 100, 70 and 45 μm filters. Immune cells were negatively selected with CD16/CD32-coated dishes (BD#553142). Supernatant containing pneumocytes was maintained with F12 (GIBCO#VX21765037) media containing penicillin/streptomycin, 2% FBS, ITS Supplements (SIGMA#ITSI1884), 0.8 mM CaCl_2_, 15 mM HEPES, 0.25% BSA. Purity of the batch was confirmed by immunofluorescence staining (Santa Cruz#13979, 1:100) and nine different high-power fields were quantified by Tissuequest. Forty-eight hours after isolation, cells were infected with calcium precipitated adenoviral particles (University of Iowa, Gene Transfer Vector Core) with a multiplicity of infection (MOI) of 250. Stimulation experiments were performed 120 h post Adenoviral Cre infection.

### Short hairpin-mediated knockdown

Lentivirus production and subsequent transduction of cells was carried out as described earlier^67^. The following shRNA constructs (Sigma Aldrich mission TRC library) were used: shRNA against STAT3 (TRCN0000071456) and control scrambled shRNA (SHC002). The functionality of the shRNAs was validated by immunoblot. Transduced cells were selected for puromycin resistance before further analysis. Within a different approach we used two *STAT3*-specific shRNAs (#336, #946) and a control shRNA (#713) and cloned them into a retroviral tetracycline-inducible expression vector^68^ containing the optimized miR-E backbone^69^. Retroviral production and subsequent transduction of cells were performed as described earlier^68^. Transduced cells were selected for neomycin resistance and doxycycline was used to induce shRNA expression. Cells were sorted for green fluorescent protein and dsRed prior usage. Short hairpin-mediated knockdown of *IL-8* was performed by cloning IL-8-specific shRNA (#1255) into a lentiviral expression vector containing the optimized miR-E backbone^69^. Lentiviral production, transduction and selection with neomycin were performed as described^69^. Cells were sorted for green fluorescent protein in addition to antibiotic selection prior usage.

### Chromatin immunoprecipitation

Chromatin isolation was done according to previously described protocol^70^. Briefly, 5 × 10^6^ A549 cells were harvested and fixed in 2 mM Di-succinimidyl Glutarate (Sigma #80424) for 45 min at room temperature. After washing, fixation with 1% formaldehyde was done for 7 min and stopped with 2.5 M glycine. Chromatin was washed and sonicated in lysis buffer (20% SDS, 0.5 M EDTA, 1 M Tris (pH 8.1), 50 × Proteinase Inhibitor Complete (Roche#11697498001), 1,000 × phenylmethylsulphonyl fluoride, 100 × Na_3_Vo_4_). Samples were sonicated for 25 cycles at 4 °C; each sonication cycle was for 30 s followed by a 30-s pause. 1% of the chromatin supernatant was kept as input. Immunoprecipitation (IP) was performed with 100 μg chromatin incubated with 1:100 diluted antibody (p65#8242, STAT3#9134 both Cell signaling, IgG control#2027 Santa cruz) for 1 h on ice. Fifty microlitres of pre-cleared beads (Life Technolgies#10004D) were added per IP and incubated rolling overnight at 4 °C. Next day, chromatin-bead complexes as well as chromatin input were washed with RIPA, high salt, lithium chloride and two times TE buffer before chromatin complexes were eluted in elution buffer (10%SDS, 0.1 M NaCO_3_, 10 mM dithiothreitol) for 30 min at room temperature. Reversing the chromatin crosslink was done with 4 M NaCl at 65 °C for more than 4 h. The IP complexes were harvested overnight at 55 °C by adding 0.5 M EDTA, 1 M Tris (pH 6.5) and Proteinase K (10 mg ml^−1^). Chromatin-DNA was isolated with Phenol/Chloroform/Isoamyl Alcohol mixture (Invitrogen#15593-031) according to protocol. DNA was subjected to quantitative reverse transcription–PCR (qRT–PCR; Kappafirst Peqlab#07KK600-03) and amount of amplification quantified with standard curves. Primer pairs are listed in Supplementary Table 1.

### RNA and real-time quantitative PCR

QRT–PCR was performed on TRIzol (Invitrogen#15596018) or Qiagen (#74104) isolated RNA. Complementary DNA transcription was performed either with a Fermentas (#K1632) or BioRad Kit (#1708890). qRT–PCR was performed using SYBR Green. Values were normalized to either murine or human *ACTB* mRNA expression. Primer pairs are listed in Supplementary Table 1.

### IP and immunoblotting

Protein lysates and nuclear/cytoplasmic extracts were done according to standard methods. IP was performed overnight with specific antibodies (STAT3 #9139, p65 #8242, Cell Signaling) and protein A Sepharose Beads (G&E#71709000AE). Immune complexes were dissolved by SDS–polyacrylamide gel electrophoresis and immunoblots were subjected with respective antibodies. Densitometric quantification of immunoblots was performed with the ImageJ software (Rasband, W.S., ImageJ, US National Institutes of Health, Bethesda, Maryland, USA, http://imagej.nih.gov/ij/, 1997–2011.) according to the standard procedures. Cytoplasmic contaminations in the nuclear fraction were corrected using the respective TUBA1A signal before nuclear extract signals were normalized to the PARP-loading control. PARP (#9532 Cell Signaling), TUBA1A (Santa Cruz#8035) or HSC-70 (Santa Cruz#7298) were used as loading controls. Supplementary Fig. 6g shows uncropped immunoblots from Fig. 5d.

### Statistics

All values are given as means±standard error of the mean (s.e.m.) or standard deviation (s.d.) as indicated. Comparisons between two groups were made either by Student’s *t*-test or Mann–Whitney *U*-Test; for more than two groups’ one-way analysis of variance (ANOVA) with Tukey’s multiple comparison test or Kruskal–Wallis test with Dunn’s multiple comparison test was performed. Tumour growth was analysed with two-way analysis of variance and Bonferroni multiple comparison testing. For the Kaplan–Meier analysis a log-rank test was performed. *P*<0.05 was accepted as statistically significant. For all graphs: *P<0.05; **P<0.01; ***P<0.001.

## Author contributions

B.G. designed and performed most experiments. J.S., L.B. performed *in situ* hybridization. K.M.M., E.B., K.Z., N.H., P.S., E.Bo., H.N. and J.S. provided major technical support. W.G., F.A., T.H. and J.Z. provided and helped with shRNA knockdown. R.H.Z. and H.P. provided major histological support. H.P. provided human data sets. H.E. and T.M. helped in microarray and gene set enrichment analysis. D.S., K.M.M., G.E., B.Gy., H.E., R.E. and H.P. assisted in data interpretation. All other co-authors gave scientific support. E.C. coordinated the project. Manuscript was written by B.G., K.M.M, H.P., H.P.M., D.S. and E.C.

## Additional information

**Accession codes:** Microarray data and description of experimental design are deposited under GEO number GSE52798. GSE29013 and GSE31210 were used for acquiring data from Fig. 1g and Supplementary Fig. 2c. In Fig. 1h,i and Supplementary Fig. 2d,e, GSE30219 and data generated by the TCGA Research Network: http://cancergenome.nih.gov/ were used.

**How to cite this article:** Grabner, B. *et al*. Disruption of STAT3 signalling promotes KRAS-induced lung tumorigenesis. *Nat. Commun.* 6:6285 doi: 10.1038/ncomms7285 (2015).

## Supplementary Material

Supplementary Information Supplementary Figures 1-6 and Supplementary Table 1
