# Supplementary material for: Disruption of STAT3 signalling promotes KRAS-induced lung tumorigenesis
Source: Nat Commun. 2015 Mar 3;6:6285. doi: 10.1038/ncomms7285 (PMC4366489; doi:10.1038/ncomms7285)
Supplement: Supplementary Information — Supplementary Figures 1-6 and Supplementary Table 1 [file ncomms7285-s1.pdf]

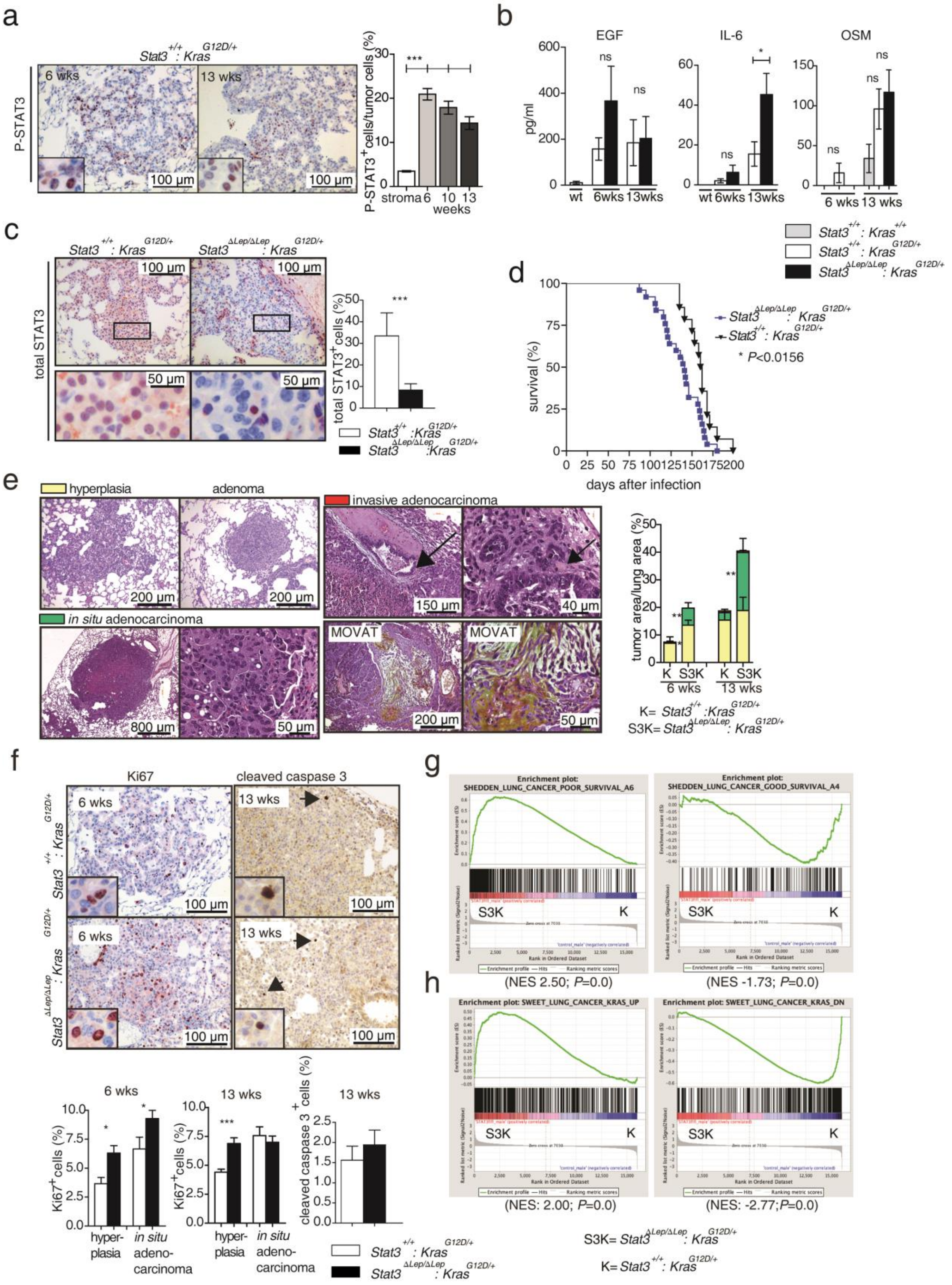

Supplementary Figure 1: STAT3 suppresses *Kras*-induced lung tumorigenesis

(**a**) Immunohistochemical (IHC) analysis of tyrosine 705 phosphorylation status of STAT3 (P-STAT3) in tumors and stroma (all-time points) of *Stat3*$^{+/+}$:*Kras*$^{G12D/+}$ mice at indicated time points. Data were analyzed by One-way Anova with Tukey's multiple comparison tests and shown as mean± s.e.m. Scale bar 100 µm. n≥ 10 tumors per mouse and n≥ 4 mice/group. (**b**) ELISA analysis of murine EGF, OSM and IL-6 in bronchioalveolar lavage (BAL) at indicated time points. Data were analyzed by One-way Anova with Tukey's multiple comparison tests and indicated as mean± s.e.m. (**c**) Representative quantification of total STAT3 (N-terminal antibody) positive cells per total tumor cells of all tumors at 10 weeks is shown. Confirmation of STAT3 deletion was done in all experiments. Data were analyzed by Student's t-test and are shown as mean ± s.e.m. Scale bar of upper image 100 µm, lower image 50 µm. (**d**) Kaplan Meier survival analysis of female mice. n≥ 14 mice/group (*P*<0.02, Log rank test)(**e**) Tumor grades were shown as following: hyperplasia (yellow, scale bar 200 µm), *in situ* adenocarcinomas (green, scale bar left 800 µm, right 50 µm) and invasive adenocarcinomas (red, scale bar 150 µm). Invasion into blood or lymphatic vessels is indicated by arrows. Desmoplastic stroma was confirmed by MOVAT staining, scale bar left 200 µm, right 50 µm. Tumor area analysis in relation to tumor grade at indicated time points shown on right; hyperplasia (yellow), *in situ* adenocarcinoma (green), invasive adenocarcinoma (red). Data were analyzed by Student's t-test and are shown as mean ± s.e.m, n≥ 7 mice/genotype. (**f**) IHC analysis of Ki67^{+} cells/ tumor cells (%) and cleaved caspase 3^{+} cells/tumor cells (%) was quantified from 3-5 tumors/mouse post AdCre n≥5 mice/genotype. Data were analyzed by Student's t-test and are shown as mean ± s.e.m. Scale bar 100 µm. (**g** and **h**) Gene Set enrichment analysis (GSEA) was performed comparing microarray profile of *Stat3*$^{\Delta Lep/\Delta Lep}$:*Kras*$^{G12D/+}$ to *Stat3*$^{+/+}$:*Kras*$^{G12D/+}$ pooled tumors isolated at 13 weeks post AdCre. NES, normalized enrichment score. For all graphs: * *P*<0.05; ** *P*<0.01; *** *P*<0.001.

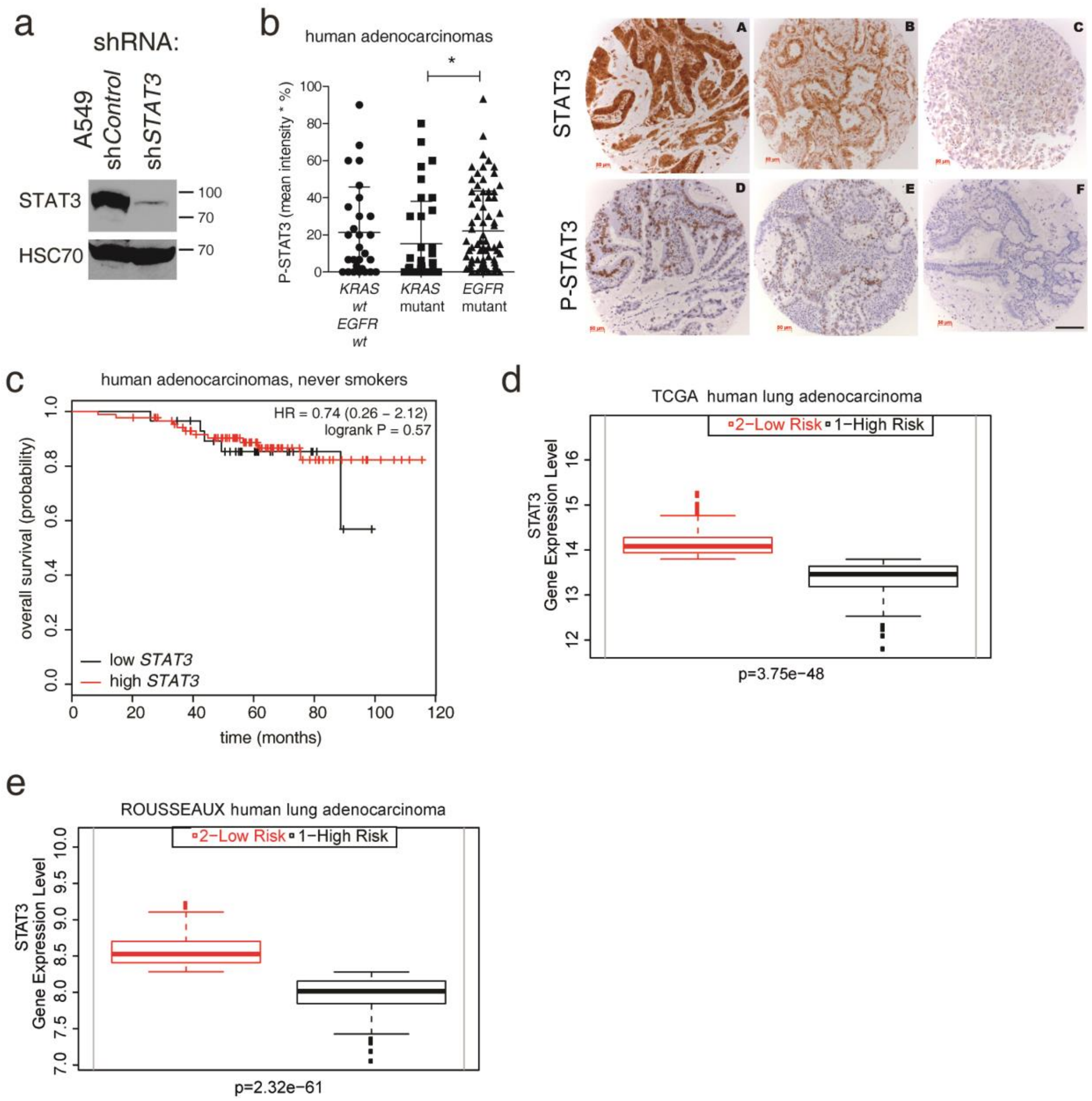

Supplementary Figure 2: STAT3 suppresses *Kras*-induced lung tumorigenesis

(a) Immunoblot validation of STAT3 knockdown in unstimulated A549 cells either transfected with lentiviral vectors expressing non-specific scrambled small hairpin RNA (sh*Control*) or shRNA against *STAT3* (sh*STAT3).* (**b**) Quantification of P-STAT3 positive staining of human lung adenocarcinoma tumor cells (n ≥ 28/group). Data were analyzed by Kruskal-Wallis test with Dunn's multiple comparison testing and shown as mean± s.d. Representative antibody reaction for STAT3 (A-C, upper panel) and for P-STAT3 (D-F, lower panel). A: strong nuclear and cytoplasmic reaction for non-phosphorylated STAT3 protein in an adenocarcinoma; B: mild positive reaction for STAT3; C: negative reaction for STAT3 – note few positive

inflammatory cells between the tumor cells; D: strong nuclear reaction for P-STAT3 in an adenocarcinoma; E: mild positive nuclear reaction for P-STAT3; F: negative reaction for P-STAT3; Scale bar 100 µm.(**c**) Kaplan-Meier curve showing overall survival of lung adenocarcinoma patient samples without smoking history with high or low *STAT3* expression (expression values of lower quartile, log rank, P=0.86). Relative *STAT3* mRNA expression levels in human lung adenocarcinoma samples. Data were extracted from (**d**) TCGA and (**e**) Rousseaux cohort database, respectively and was classified into high and low risk groups. Data are shown as box plots including the p value calculated by Student's t- test. For all graphs: * $P<0.05$;

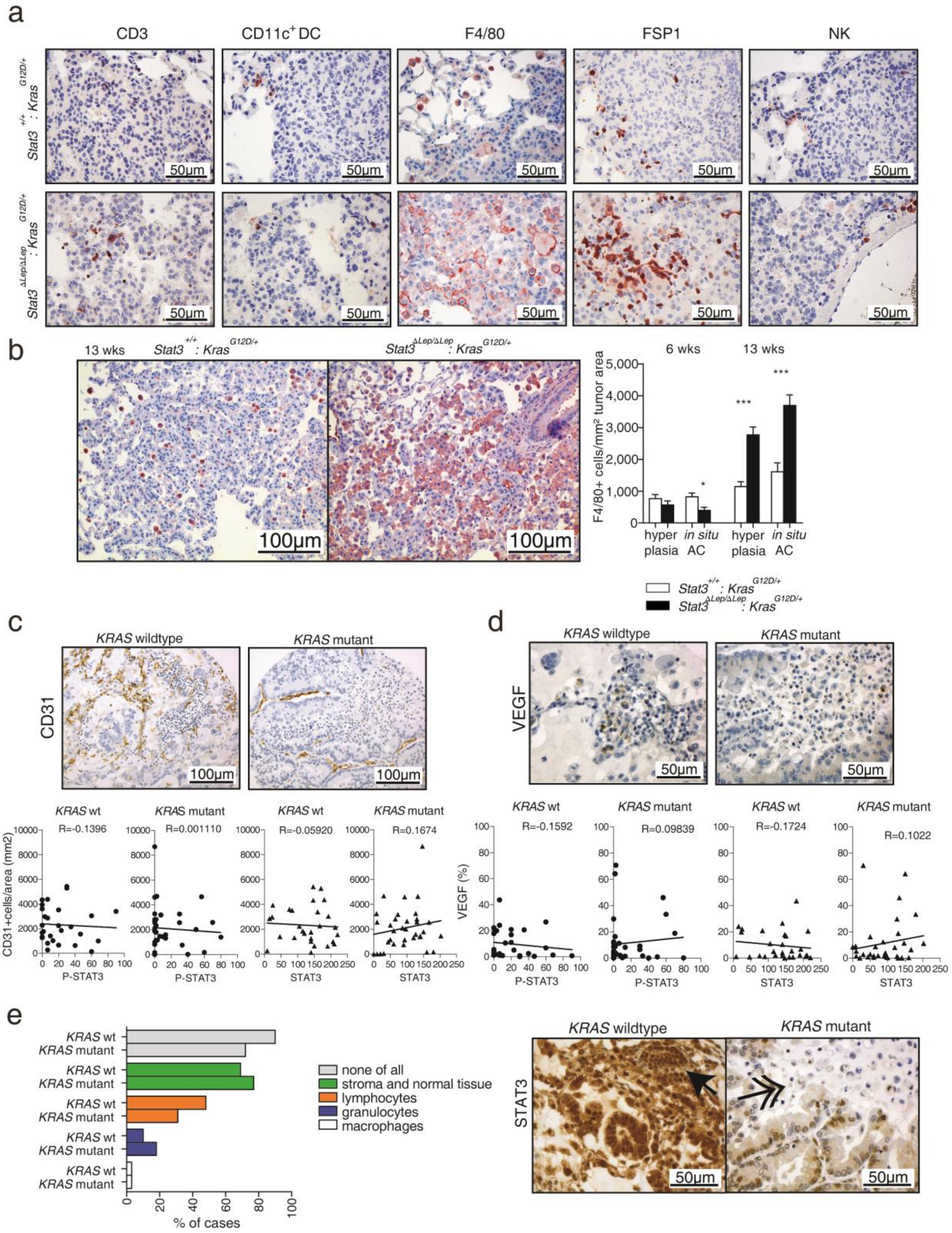

Supplementary Figure 3: STAT3 alters tumor microenvironment and angiogenesis

(**a**) IHC pictures of CD3+ T cells, Cd11c+ dentritic cells (DC), F4/80+ macrophages, FSP1+ fibroblasts and NKp46+ NK cells present within lung tumors. Scale bar 50 µm. (**b**) IHC analysis of F4/80^{+} cell counts per tumor area (mm²). Data are shown as mean ± s.e.m with

n ≥ 5 tumors/mouse and n ≥ 4 mice/genotype and time point. Scale bar 100 µm.**P*<0.05, ****P*<0.001 as analyzed by Student's t-test (**c**) Representative CD31 staining of human patient samples (*KRAS* wildtype versus *KRAS* mutant). Scale bar 100 µm. Correlation analysis between CD31 and STAT3 or P-STAT3 in *KRAS* wildtype versus *KRAS* mutant human samples is shown. R value indicates Spearman Correlation Coefficient. (**d**) Representative VEGF staining of human patient samples (*KRAS* wildtype versus *KRAS* mutant). Scale bar 50 µm. Correlation analysis between CD31 and STAT3 or P-STAT3 in *KRAS* wildtype versus *KRAS* mutant patient samples is shown. R value indicates Spearman Correlation Coefficient. (**e**) Qualitative assessment of tumor infiltrates in *KRAS* wildtype versus *KRAS* mutant patient samples. Representative immunostaining of STAT3 in *KRAS* wildtype and *KRAS* mutant tumors are shown. Arrow on STAT3 high expressing sample (left) indicates lymphocyte infiltrates, and arrows on STAT3 low expressing sample (right) indicate granulocyte infiltrates. Scale bar 50 µm.

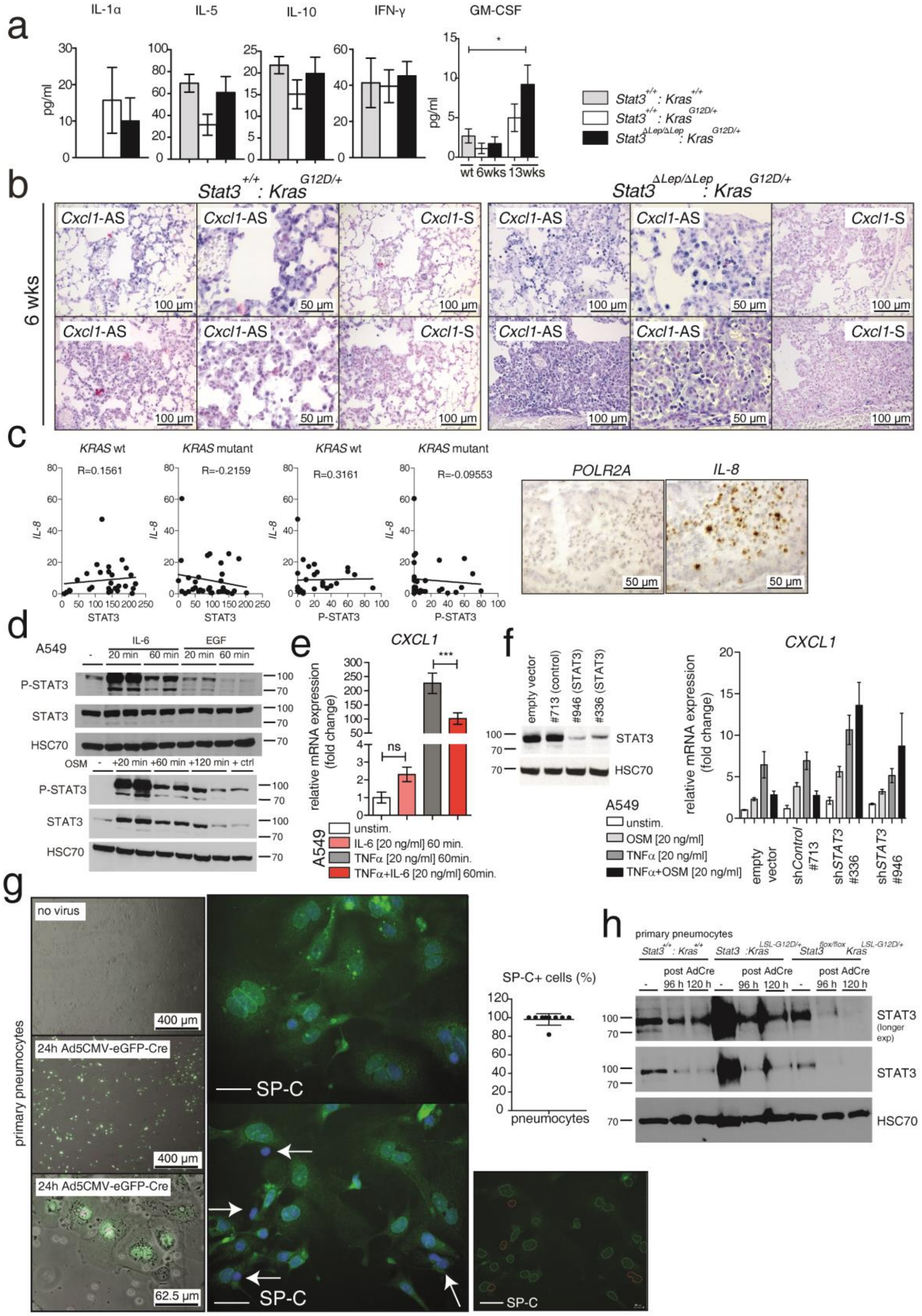

Supplementary Figure 4: STAT3 regulates chemoattractive CXCL1 expression

(**a**) ELISA analysis of indicated cytokines collected from bronchoalveolar lavage at different time points (n ≥ 5 mice/genotype). Data were analyzed by One-way Anova with Tukey's multiple comparison test or Kruskal-Wallis test with Dunn's multiple comparison testing and are shown as mean ± s.e.m. (* *P*<0.05) (**b**) *In situ* hybridization of lung tumors 6 weeks post AdCre using *Cxcl1* (AS) specific probes. Corresponding sense probes [*Cxcl1* (S)] served as negative control. Scale bar as indicated. (**c**) Correlation analysis of *IL-8* mRNA expression with STAT3 or P-STAT3 levels in *KRAS* mutant vs *KRAS* wildtype patient samples. R indicates Spearman correlation coefficient. Representative pictures of *POLR2A* and *IL-8* stainings are shown. Scale bar 50 µm. (**d**) Immunoblot analysis of P-STAT3 and total STAT3 levels of A549 cells stimulated with IL-6, EGF or OSM for indicated time points. HSC70 served as loading control. (**e**) Relative mRNA expression levels of *CXCL1* in A549 cells after stimulation with indicated cytokines (60 min). Data were analyzed by One-way Anova with Tukey's multiple comparison test shown as mean ± s.d. *** *P*<0.001. (**f**) Confirmation of retroviral mediated tetracycline inducible STAT3 shRNA knockdown (#336 and #946) by immunoblot and relative mRNA expression levels of *CXCL1* in A549 cells after 60 min stimulation with indicated cytokines. Empty vector or shRNA against Luciferase (#713) were used as controls. Data in (e) and (f) were analyzed by One-way Anova with Tukey's multiple comparison and indicate mean ± s.e.m of at least two independent experiments. (**g**) Murine alveolar pneumocytes were isolated; purity of the batch was confirmed by surfactant protein-C (SP-C) immunofluorescence staining. Left panel shows viral transduction of pneumocytes by Ad5CMV-eGFP-Cre 48h post isolation with scale bar as indicated. Pictures in the middle panel show 100% of SP-C positive cells (upper) and 82% positive cells (lower), as analyzed by Histoquest (lower right panel). Quantification of 9 HPF regions is shown on the right. White arrows indicate SP-C negative cells. White size bar indicates 50 µm. (**h**) Validation of STAT3 knockout in primary pneumocytes after indicated time points by immunoblot Including HSC70 as loading control.

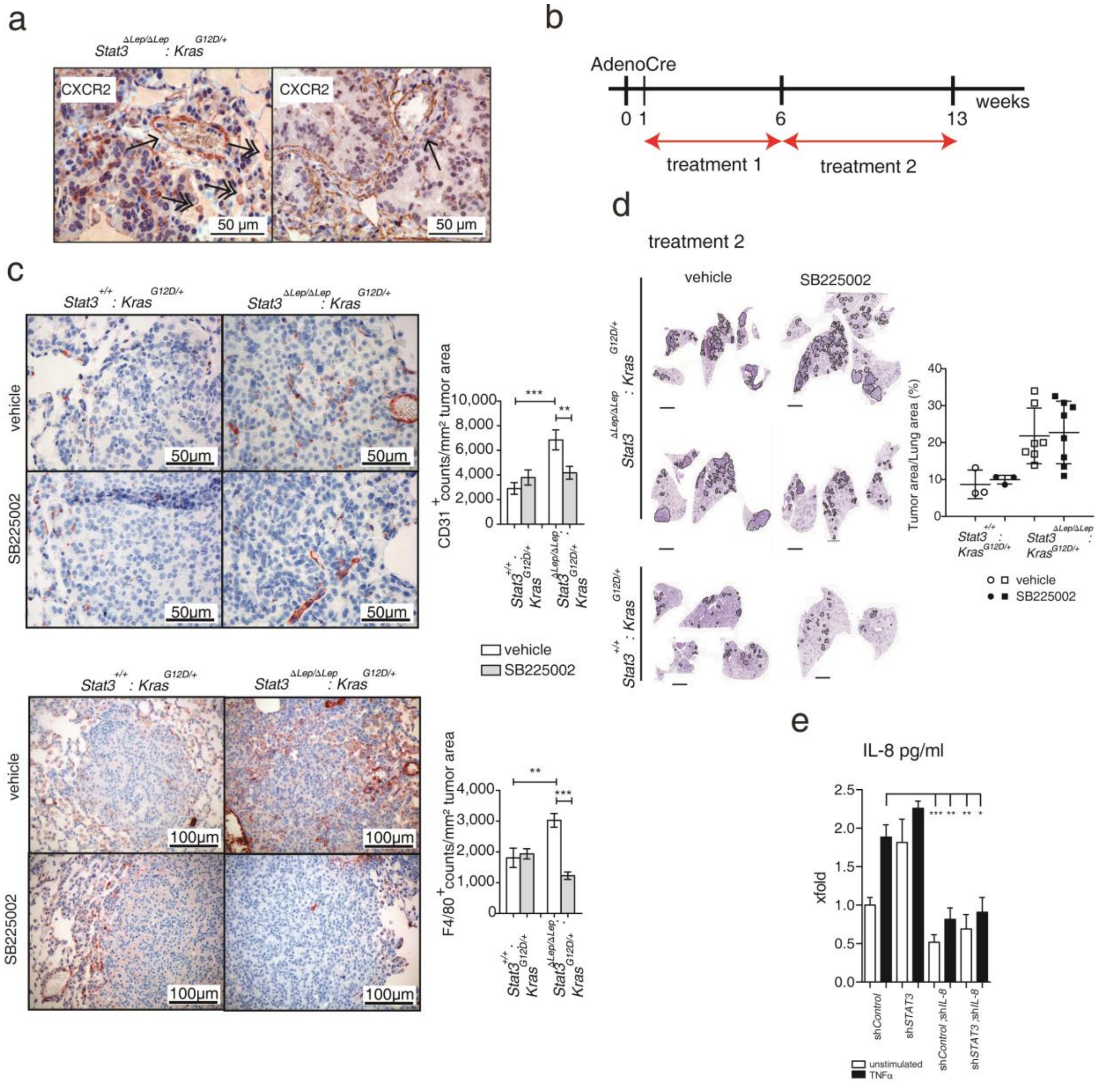

Supplementary Figure 5: CXCL1 inhibiton reverts oncogenic effects of STAT3 ablation

(**a**) Representative pictures of CXCR2 expression analysis by immunohistochemistry in *Stat3*$^{\Delta Lep/\Delta Lep}$*: Kras*$^{G12D/+}$ mice. Single arrows indicate endothelial positive staining; double headed arrows indicate positively stained macrophages; scattered epithelial cells showed immunoreactivity. Scale bar 50 µm. (**b**) Timeline of treatment approaches (5 mg/kg bodyweight for 5 days/week) or vehicle control. Treatment 1 started one week after tumor induction until 6 weeks post AdCre. Treatment 2 was initiated at 6 weeks post AdCre and performed until 13 weeks post AdCre. (**c**) Tumor vascularization was quantified by CD31+ counts per *in situ* adenocarcinoma tumor area (mm²). Tumor infiltration was quantified by

F4/80+ macrophages per in situ adenocarcinoma tumor area (mm^2). Data were analyzed by One-way Anova with Tukey's multiple comparison test and are shown as mean ± s.e.m. At least 6 tumors/mouse were analyzed with $n \geq 3$ mice/genotype and treatment. Scale bar as indicated. (**d**) Representative H&E stained lung sections of treatment 2. Tumor area/lung area was quantified within each group and at least three sections of each lung were stained with H&E and analyzed in a blinded fashion (n=3-8 mice/genotype). Data are analyzed by One-way Anova with Tukey's multiple comparison test and are shown as mean ± s.d. Scale bar 2 mm. (**e**) IL-8 ELISA of A549 cell culture supernatants. Cells were transduced with lentivirus expressing control and STAT3 shRNA, as well as a combination of IL-8 shRNA with control/STAT3 shRNA and either left unstimulated or stimulated with 20ng/ml TNFα for 8 hours. Data represents x-fold IL-8 levels compared to A549 sh*Control*. (n=3 independent wells per stimulation). Data were analyzed by One-way Anova with Tukey's multiple comparison test and are shown as mean ± s.e.m. For all graphs: *P<0.05; **P<0.01; ***P<0.001.

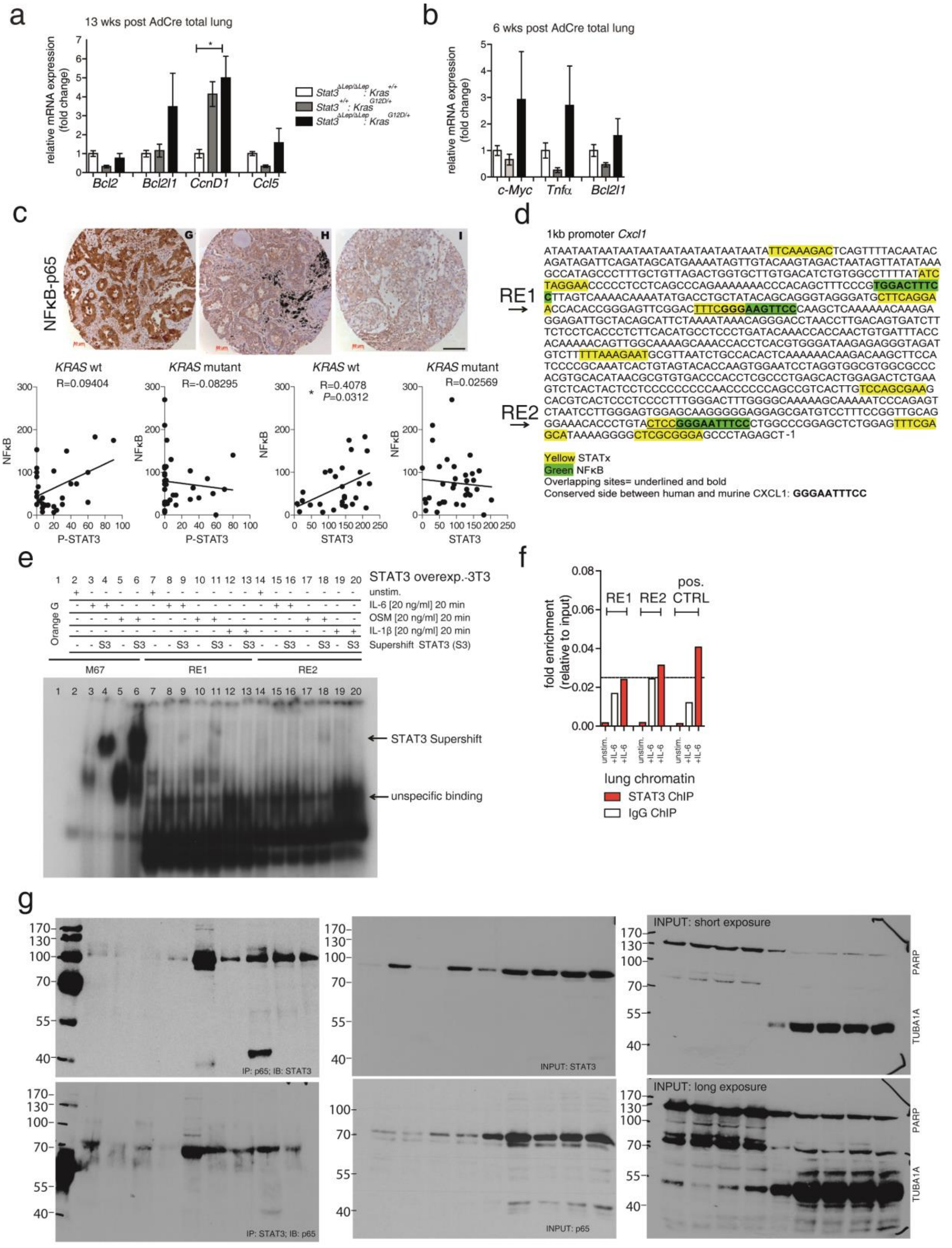

Supplementary Figure 6: STAT3 retains p65 in the cytoplasm to reduce NFκB activity.

(**a**) Relative mRNA expression levels of *Bcl2, Bcl2l1, Ccnd1, Ccl5* and (**b**) *c-Myc, Tnfα* and *Bcl2l1* were measured by qRT-PCR at indicated timepoints. Data were analyzed by One-way

Anova with Tukey's multiple comparison test and shown as mean ± s.e.m., n=6-8 mice/genotype. **P*<0.05. (**c**) Representative pictures of (G) strong positive nuclear and cytoplasmic reaction for NFκB in mucinous adenocarcinoma; (H) mild cytoplasmic reaction for NFκB (black staining is tar); (I) focal and minimal positive reaction for NFκB is shown. Scale bar 100 µm. Correlation analysis between NFκB subunit p65 and STAT3 or P-STAT3 expression levels in *KRAS* mutant versus *KRAS* wildtype patients was performed. R indicates Spearman correlation coefficient. STAT3 and NFκB subunit p65 expression display a significant positive correlation in *KRAS* wildtype patients (P=0.0312). (**d**) 1kb of the murine *Cxcl1* promoter sequence upstream of transcriptional start site is shown. Putative NFκB binding sites are shown in green and putative STAT binding sites in yellow. Overlapping NFκB and STAT binding sites are underlined. Conserved sites between murine and human *CXCL1* promoter are shown in bold. (Responsive Elements, RE) (**e**) Putative binding sites RE1 and RE2 were analyzed by electrophoretic mobility shift assay (EMSA). STAT3-overexpressing 3T3 cells were stimulated with murine IL-6, OSM or IL1β for 20 minutes. (**f**) Chromatin-IP (ChIP) analysis of putative binding sites *in vivo*. C57BL6/N mice were injected intraperitoneally with 40 ng/body weight murine IL-6 or mock control. Lungs were harvested 30 minutes post injection. ChIP of lung lysates was performed using STAT3 antibody and IgG isotype control. Binding of STAT3 to *Socs3* promoter served as positive control. As negative binding region a *Junb* element was chosen, indicated by the horizontal line. Values are presented as fold enrichment relative to the input chromatin. (**g**) Whole membranes of immunblots against STAT3, p65, PARP and α-tubulin are shown (compare Fig 5d.).

SUPPLEMENTARY TABLE 1. Primer list

qPCR primer list	
c-Myc fw	ACGGAGTCGTAGTCGAGGTC
c-Myc rev	AGAGCTCCTCGAGCTGTTTG
Actb fw	GCTCATAGCTCTTCTCCAGGG
Actb rev	CCTGAACCCTAAGGCCAACCG
Pdgfa fw	CCTCACCTGGACCTCTTTCA
Pdgfa rev	TAACACCAGCAGCGTCAAGT
Tnfa fw	ATGAGAGGGAGGCCATTTG
Tnfa rev	CAGCCTCTTCTCATTCCTGC
Ccl5 fw	CCACTTCTTCTCTGGGTTGG
Ccl5 rev	GTGCCCACGTCAAGGAGTAT
Ccnd1 fw	GGGTGGGTTGGAAATGAACT
Ccnd1 rev	CTTCCTCTCCAAAATGCCAG
Bcl2 fw	ACGGAGGCTGGGATGCCTTT
Bcl2 rev	AGTGATGCAGGCCCCGACCA
Bcl2l1 fw	CGGGGCACTGTGCGTGGAAA
Bcl2l1 rev	AAGTGTCCCAGCCGCCGTTC
Cxcl1 fw	TCTCCGTTACTTGGGGACAC
Cxcl1 rev	CCACACTCAAGAATGGTCGC
ACTB fw	GCACAGAGCCTCGCCTTTGCC
ACTB rev	CATGCCCACCATCACGCCCTGG
CXCL1 fw	AACAGCCACCAGTGAGCTTC
CXCL1 rev	GAAAGCTTGCCTCAATCCTG
IL-8 fw	AGCACTCCTTGGCAAAACTG
IL-8 rev	CGGAAGGAACCATCTCACTG
STAT3 fw	TGACATTCCCAAGGAGGAGGC
STAT3 rev	TGCAGCTTCCGTTCTCAGCTCC
EMSA primer list	
M67 fw	CATTTCCCGTAAATC
M67 rev	GATTTACGGGAAATG
Cxcl1 Re1 fw (murine)	GACTTTCGGGAAGTTCCCAAGC
Cxcl1 Re1 rev (murine)	GCTTGGGAACTTCCCGAAAGTC
Cxcl1 Re2 fw (murine)	GTACTCCGGGAATTTCCCTGGC
Cxcl1 Re2 rev (murine)	GCCAGGGAAATTCCCGGAGTAC
ChIP primer list	
ICAM1 fw	ACCGTGATTCAAGCTTAGCC
ICAM1 rev	TGCAGTTATTTCCGGACTGA
ACTB fw	CCTGCAGAGTTCCAAAGGAG
ACTB rev	AAGATGACCCAGGTGAGTGG
Cxcl1 Re1 fw (murine)	TACAGCAGGGTAGGGATGCT
Cxcl1 Re1rev (murine)	TGTATCAGGGAGGCATGTGA
Cxcl1 Re2 fw (murine)	TAATCCTTGGGAGTGGAGCA
Cxcl1 Re2 rev (murine)	GGAGTCTGGAGTGCTGGAAC
CXCL1 RE2 fw (human)	ATCAGTGGACCCCCACAC
CXCL1 RE2 rev (human)	ACCCCTTTTATGCATGGTTG
